# Study on fresh and hardened state properties of eco-friendly foamed concrete incorporating waste soda-lime glass

**DOI:** 10.1038/s41598-024-69572-4

**Published:** 2024-08-12

**Authors:** Md Azree Othuman Mydin, P. Jagadesh, Alireza Bahrami, Samadar S. Majeed, Anmar Dulaimi, Roshartini Omar

**Affiliations:** 1 School of Housing, Building and Planning, Universiti Sains Malaysia, 11800 Penang, Malaysia; 2 Department of Civil Engineering, Coimbatore Institute of Technology, Coimbatore, Tamil Nadu 638 056 India; 3 Department of Building Engineering, Energy Systems and Sustainability Science, Faculty of Engineering and Sustainable Development, University of Gävle, 801 76 Gävle, Sweden; 4 Civil Engineering Department, College of Engineering, Nawroz University, Duhok, Kurdistan Region Iraq; 5 Department of Civil Engineering, College of Engineering, University of Kerbala, Karbala, 56001 Iraq; 6 College of Engineering, University of Warith Al-Anbiyaa, Karbala, 56001 Iraq; 7 Department of Construction Management, Faculty of Technology Management and Business, Universiti Tun Hussein Onn Malaysia, 86400 Parit Raja, Batu Pahat, Johor Malaysia

**Keywords:** Foamed concrete, Waste soda-lime glass bottle, Sand replacement, Compressive strength, Thermal conductivity, Permeable porosity, Scanning electron microscopy, Engineering, Civil engineering

## Abstract

Improper waste management is causing global environmental problems. Waste glass may have adverse impacts on the ecosystem. While a substantial amount of soda-lime glass bottle (SGB) undergoes recycling to create new glass items, a significant volume still ends up in landfills. Therefore, the aim of this study was to explore the potential use of SGB in foamed concrete (FC) production as an aggregate replacement. SGB was substituted for sand in different weight fractions, ranging from 5 to 50%. The fresh state, mechanical, thermal, pore structure, and transport properties were examined. The findings showed a significant enhancement in the FC’s mechanical properties when SGB replaced 20% of sand. The compressive, flexural, and splitting tensile strengths exhibited a rise of up to 17.7, 39.4, and 43.8%, respectively. The findings also demonstrated that the addition of SGB improved the thermal conductivity, sorptivity, water absorption, and porosity. The scanning electron microscopy analysis indicated that the inclusion of 20% SGB caused a substantial decrease in void diameter and enhanced its uniformity. A comparison was made between the experimental data and predictions of the mechanical properties using various models of international standards, such as IS 456, ACI 318, NZS-3101, EC-02, AS 3600, and CEB-FIB, along with several references in the literature. The findings implied a strong correlation between the strength properties. The outcomes of this research offer valuable insights into both the possible advantages and constraints of using SGB in FC. Furthermore, this extensive laboratory investigation may serve as a guideline for future study and aid in the advancement of greener and more environmentally friendly FC alternatives.

## Introduction

Foamed concrete (FC) technology has gained considerable interest in recent years due to its low density, exceptional thermal and sound insulation characteristics, and excellent earthquake and fire protection capabilities^1–3^. In addition, researchers have investigated the possibility of utilizing FC as materials for adsorption, 3D-printing, and photocatalysis. These innovations have illustrated to decrease construction costs and time by eliminating the need for formwork and minimising on-site labour^4–6^. FC is created by introducing air voids that are randomly distributed, resulting in a reduced density ranging from 300 kg/m^3^ to 1850 kg/m^3^^7^. Foam is generated as the outcome of a chemical reaction, which subsequently leads to the formation of further pores in the cement slurry^8,9^. This technique aims to reduce the self-weight of FC. The increased porosity of FC is a crucial factor that results in a decrease in its own weight^10^.

Proteins and synthetics are frequently employed as foaming agents in the manufacturing of FC^11^. The microstructures formed by protein-based foaming agents are robust and stable compared to those formed by other types of foaming agents^12^. A number of scholars have directed their research towards synthetic foaming agents, as evidenced in the literature^13–15^. FC can be produced by either mixed foaming or pre-foaming methods. Regardless of the selected method for creating foam, it is essential for the foam to exhibit the stability and solidity. It is essential for the material to be able to resist the force applied by cement mortar and the strong structure surrounding the pores in FC^16^. The commonly used FC foaming agents include hydrogen peroxide, carbide, and aluminium powder^17^.

Researchers, engineers, and academicians have devised novel solutions that consider both environmental protection and economic conditions to attain a minimum FC’s compressive strength of 25 MPa^18^. Jalal et al.^19^ reported that FC possessed adequate strength to serve as a construction material, eliminating the need for compaction to fill the cavities. In addition to its exceptional freezing and thawing characteristics, FC displayed remarkable fire resistance. Similar to conventional concrete, the production of FC using natural resources results in several disadvantages, such as pollution and resource utilization. Therefore, it is imperative to employ waste materials in the manufacture of FC, as this promotes sustainability in the production process. Literature has demonstrated the replacement of binder with pozzolanic materials like palm oil fuel ash (POFA), rice husk ash (RHA), and fly ash (FA). For example, the use of FA as binder provides the enhanced mechanical and durability, as reported by several studies^20–23^. Additionally, researchers are using RHA as a partial binder replacement to enhance the strength and thermal characteristics of FC^24^. Further studies are underway on upcoming pozzolanic materials like POFA to make FC a sustainable product^25,26^.

Many countries are currently undergoing a rapid expansion of their construction sector, driven by the utilization of natural resources to build infrastructure and skyscrapers. It is crucial to recognize that reducing carbon dioxide emissions and protecting biodiversity are two major issues that modern society encounters^27^. Concrete production, which is an important factor contributing to carbon dioxide releases and gradual dwindling of natural resources, accounts for approximately 10% of worldwide greenhouse gas emissions and 30% of non-renewable natural resource consumption^28^. This development may be imperilled due to the limited availability of easily obtainable natural resources. Globally, both the quantity of waste produced by industries and depletion of natural resources are increasing significantly^29^.

Most studies utilize natural fine aggregate (NFA) in FC, primarily consisting of silicon dioxide (SiO_2_) from natural river sand. The physical characteristics of NFA include a uniform granular shape and consistency on its surface. According to Gencel et al.^24^, recyclable fine aggregates have lower thermal conductivity and more permeable porosity. They also have lower dynamic elastic modulus. Xiao et al.^30^ found that replacing NFA with recycled fine aggregate (RFA) and replacing cement with recycled powder increased the fluidity, decreased the compressive strength, and enhanced the energy absorption. By replacing NFA in FC with POFA, transport properties, microstructural, and strength attributes enhanced, shrinkage properties were increased, and sulphate resistance was decreased^31^. Replacing NFA with metakaolin improved the strength performance, density, and abrasion resistance. Table 1 summarizes studies on FC with NFA substitution by waste materials and their impact on FC characteristics.

**Table 1:** FC with replacement of fine aggregate.

Reference	Materials used for replacement of fine aggregate	Evaluated properties	
		Fresh concrete	Hardened concrete
Akhund et al.^32^	Recycled biomass aggregate	Density	Compressive strength (6.3 MPa to 8.2 MPa)
Lim et al.^26^	Quarry waste	Flow table test, density	Conductivity, compressive strength (5.68 MPa to 6.46 MPa, 6.15 MPa to 6.85 MPa), and void size distribution
Ikponwosa et al.^33^	Polyvinyl waste	Density	Compressive strength (3.00 MPa to 15.00 MPa)
Sharipudin and Ridzuan^34^	RFA		Compressive strength (9.5 MPa to 14.5 MPa)
Hadipramana et al.^35^	RHA	Density	Compressive strength (1.8 MPa to 10.5 MPa) and microstructure studies
Hajimohammadi et al.^36^	Glass fines	Density	Compressive strength (2.4 MPa to 3.1 MPa), shrinkage test, pore morphology, and thermal properties
Maglad et al.^37^	Glass sheet powder	Density	Compressive strength (4.8 MPa to 7.2 MPa), splitting tensile strength (0.8 MPa to 1.2 MPa), flexural strength (1.4 MPa to 1.9 MPa), conductivity, diffusivity, and specific heat capacity
Selvakumar et al.^38^	Foundry sand	Density	Compressive strength (8.87 MPa to 10.12 MPa), flexural strength (1.32 MPa to 1.56 MPa), sorptivity, and chloride penetration
Shwetha et al.^39^	Granite waste powder	Density	Water absorption, flexural strength (2.42 MPa to 2.94 MPa), compressive strength (32.40 MPa to 41.22 MPa), and conductivity

Given the global increase in concrete production, it is vital to explore the substitution of elements in concrete, such as aggregates, with waste materials. One specific waste material found in this investigation is waste soda-lime glass bottles (SGBs). Glass waste is accumulated from numerous sources and discarded or reclaimed to fulfil diverse needs^40^. Waste glass aggregates have a substantial impact on the concrete strength. By replacing aggregates with waste glass, concrete has higher flexural and compressive strengths. Waste glass serves as an alkali activating agent in the production of geopolymer FC^41,42^. Foam glass-based aggregate, characterized by its low density, contributes to the production of ultra-lightweight concrete. This type of concrete has exceptional resilience to high temperatures and has a reduced ecological impact. Moreover, it indicates the increased compressive strength and improved resistance to the thermal conductivity^43^. Compared to other types of glass, concrete produced with soda-lime glass has increased the flexural and compressive strengths because of its high silica content and rough surface^44,45^. It has been shown that using waste glass as filler in concrete manufacturing improves its freshness, mechanical properties, and durability^46–49^. Glass powder has displayed to improve the concrete’s characteristics when it is substituted for some or all the fine and coarse aggregates in several studies^50,51^.

Rajabipour et al.^52^ examined the possibility of the alkali silica reaction (ASR) in the larger soda lime glass aggregates compared to the smaller soda lime glass aggregates. Additionally, they observed that an increase in the surface area of the soda lime glass aggregates diminished the ASR response. A scanning electron microscopy (SEM) investigation of the ASR-affected glass particles revealed that they do not undergo an ASR reaction at the surface. However, ASR triggered a fracture within the intra-particles formed during the crushing process^52^. Shafaatian et al.^53^ reported that adding pozzolanic materials to glass aggregate concrete lowered the ASR reaction because calcium silicate hydrate (CSH) formed at the point where the glass aggregates met the mortar paste.

From the above review, the majority of previous studies have concentrated on the utilization of glass sheet powder, which is acquired as waste materials from construction industries, particularly in areas such as window glass covering and elevation applications. These glasses exhibit different chemical and physical properties due to their specific intended function^37,52,53^. In this study, the waste SGB was utilized as a partial substitute for fine aggregate, serving a distinct purpose.

## Research significance

There is a scarcity of research on improving the FC’s characteristics while also considering the cost reduction, environmental protection, and natural resource preservation. Quarry sand, FA, foundry sand, and reclaimed fine aggregate are among the waste materials that can replace NFA. The current research on the NFA replacement has not thoroughly examined all possible alternatives. SGB is one of the identified wastes. The use of SGB as a replacement for fine aggregate in concrete production has significant environmental and economic benefits. The SGB consumption reduces the need for fine aggregates, preserving natural resources while minimizing the environmental consequences of their processing and extraction. Literature frequently describes SGB for its pozzolanic properties, which improve its performance in both fresh and hardened states.

## Materials and methods

### Raw materials

#### Cement

Ordinary Portland cement (OPC) grade of 52.5 N, as stated in BS197-1^54^, was used in this study. A CEM I product consists of 95% clinker and 5% small additives. The composition mostly consists of oxides such as CaO, SiO_2_, Fe_2_O3, Al_2_O_3_, and several minor components.

#### NFA

**Figure 1:**
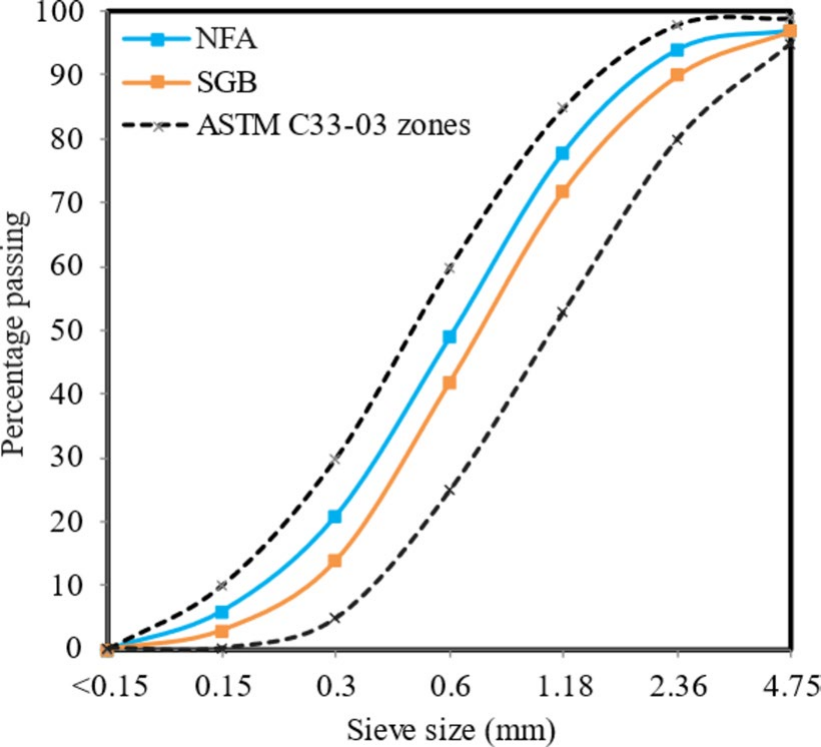
NFA and SGB particles size distribution.

NFA was acquired from a local source. It has a specific gravity of 2.54 and a surface dry specific gravity of 2.59, which aligns with ASTM C33-08^55^. To manufacture FC, it is necessary to use NFA that is less than 4.75 mm. Larger particle size can result in the deterioration of foam and may lead to segregation. The NFA particle sizes are depicted in Fig. 1.

#### Water

As per BS 3148^56^, clean water from the tap has been utilized. Water quality is essential to ensure the reliability and properties of FCs.

#### Foaming agent

The study employed Noraite PA-1, a protein-based foaming agent. It was decided to use a protein-based agent instead of synthetic-based, which was based on the results of the pilot study, which revealed that protein-based FC had superior mechanical and durability properties. Furthermore, the protein foaming agent generated stable and consistent bubble sizes. To achieve the required foam stability, it was diluted with water at a ratio of 1:25 to produce a foam density of 65 kg/m^3^.

#### Soda glass powder

**Figure 2:**
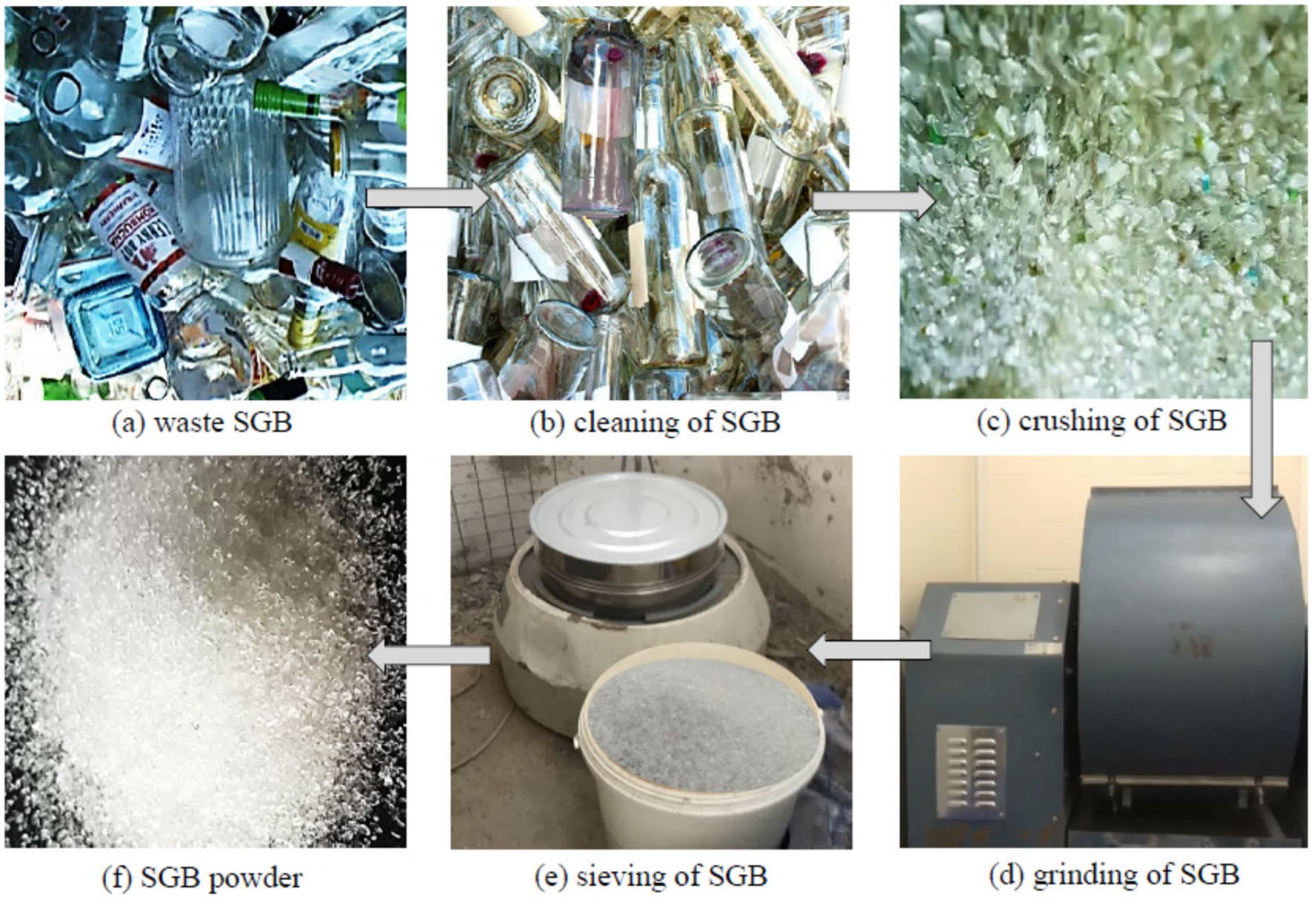
Process of producing waste SGB as fine aggregate.

**Figure 3:**
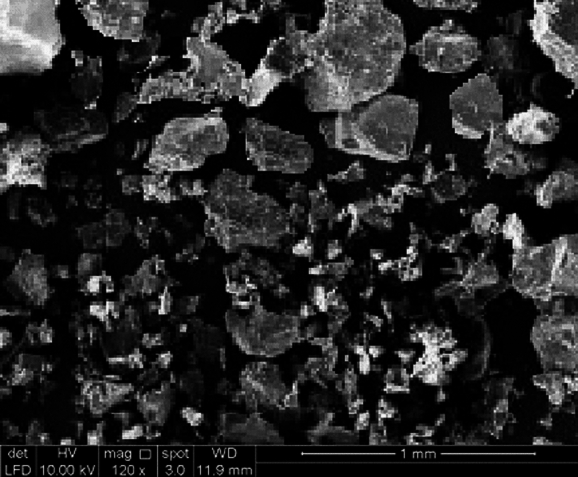
SEM image of SGB.

SGB utilized in this study possessed a bulk density of 1760 kg/m^3^, a specific gravity of 2.58, and a fineness modulus of 2.29. The waste SGBs were collected from a recycling centre. Firstly, the bottles were cleaned properly to eliminate any debris and impurities. Then, the bottles were mashed into small pieces of about 10 mm. Next, these small pieces of glass were ground to form a glass powder using a Los Angeles grinding machine. Afterwards, the glass was manually sifted to achieve a particle size that was similar to or less than the size of the sand particles. The process of producing SGB is illustrated in Fig. 2. Fernandes et al.^57^ found that replacing NFA with SGB of the lowest particle size improved both the fresh and hardened properties of FC. Figure 1 displays the particle size distribution of SGB, which was then compared to ASTM C33-08^55^. Table 2 presents the chemical composition of OPC and SGB, which was analysed utilising X-ray fluorescence with a Philips Ibérica Magic Pro spectrometer device. Figure 3 exhibits the morphological characteristics of SGB considered in this study. Porous structures with uneven surfaces can be seen.

**Table 2:** Chemical compositions of OPC and SGB.

Components	OPC	SGB
Calcium oxide (CaO)	60.39	9.86
Silicon dioxide (SiO_2_)	20.97	71.59
Potassium oxide (K_2_O)	0.49	0.97
Iron oxide (Fe_2_O_3_)	2.78	0.32
Magnesium oxide (MgO)	3.68	1.43
Sodium oxide (Na_2_O)	0.16	12.87
Aluminium oxide (Al_2_O_3_)	5.82	1.95
Sulfur trioxide (SO_3_)	2.89	–
Chromium (III) oxide (Cr_2_O_3_)	–	0.04
LOI	2.82	0.97

### Methods

#### Mix proportions

This research work involved the production and examination of a high-density FC with a density of 1850 kg/m^3^. The employed cement-to-sand ratio was 1:1.5, ensuring the appropriate stability between the binding element and NFA. The water-to-cement ratio was maintained at a constant value of 0.45, ensuring the optimal moisture content for the process of hydration and desired qualities of fresh concrete. To analyse the impact of SGB on FC, the authors substituted NFA with SGB in several proportions, specifically 5, 10, 15, 20, 25, 30, 35, 40, 45, and 50%. Table 3 lists the composition ratios of the FC’s combinations with SGB.

**Table 3:** FC’s mix proportions.

FC mix	SGB (%)	OPC (kg/m^3^)	Sand (kg/m^3^)	SGB (kg/m^3^)	Water (kg/m^3^)	Foam (kg/m^3^)
S0	0	681.6	1022.4	0	306.7	4.09
S5	5	681.6	971.3	51.1	306.7	4.09
S10	10	681.6	920.2	102.2	306.7	4.09
S15	15	681.6	869.1	153.4	306.7	4.09
S20	20	681.6	817.9	204.5	306.7	4.09
S25	25	681.6	766.8	255.6	306.7	4.09
S30	30	681.6	715.7	306.7	306.7	4.09
S35	35	681.6	664.6	357.9	306.7	4.09
S40	40	681.6	613.4	409.0	306.7	4.09
S45	45	681.6	562.3	460.1	306.7	4.09
S50	50	681.6	511.2	511.2	306.7	4.09

#### Preparation of FC

**Figure 4:**
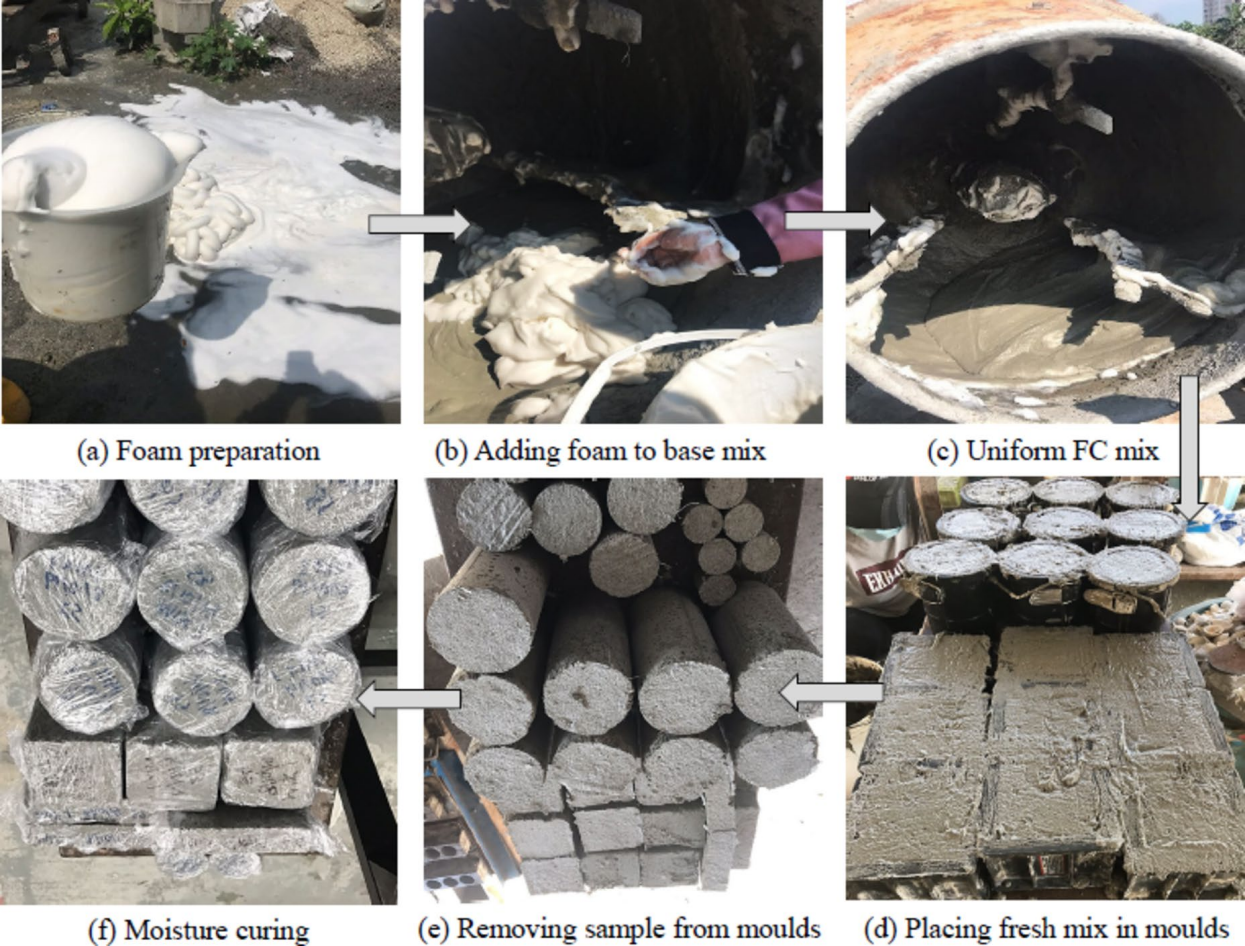
Process of manufacturing FC.

Figure 4 shows the process of manufacturing FC. FC was manufactured in two phases. In the first phase, Noraite PA-1 protein foaming agent was applied to create stable foam, as illustrated in Fig. 4a. The manufacture of stable foam was done by adding a foaming agent to water in a ratio of 1:25 and utilizing a compressor to generate foam on the Portafoam TM-2 machine as the foam generator. In the second phase, OPC, NFA, and SGB were blended for two minutes. Water was added to the dry mixture and further mixed for 4 min.

Figure 4b depicts the addition of stable foam to create the FC slurry mixture. The quantity of foam added to the base mix was according to the standard mix design calculation as follows:

$$Required\;foam = \left[ {1 - \left( {\frac{wet\;density}{{2150}}} \right)} \right] \times mix\;volume \times foam\;density$$

$$Required\;foam = \left[ {1 - \left( {\frac{2015}{{2150}}} \right)} \right] \times 1 \times 65 = 4.09\;{\text{kg}}/{\text{m}}^{3}$$

The foam output from Portafoam had a flow rate of 6.5 l/s. Therefore, it was necessary to run the generator for a duration of 629 s to inject a total volume 4.09 kg/m^3^ into the base mix.

After blending for approximately five minutes, a homogenous mix was obtained as demonstrated in Fig. 4c. The freshly FC mixture was placed in steel moulds (Fig. 4d). After a period of 24 h, the FC samples were extracted from the moulds (Fig. 4e). A moisture curing process (Fig. 4f) was followed until the FC specimens were ready for testing.

#### Experimental procedure

This study aims to examine the freshness, mechanical, transport, thermal, and microstructural properties of FC produced with the addition of SGB. The tests were conducted following international guidelines. The test name, sample form, size, and testing ages had been determined according to the specifications and are summarized in Table 4.

**Table 4:** Description of tests and specimens.

Properties	Type of test	Shape	Size (mm)	Age (day)	International standard
Freshness	Slump	–	–	Casting	ASTM C230-03^58^
	Setting time	–	–	Casting	BS 196-–3^59^
	Hardened density	–	–	Casting	BS 12350-6^60^
Mechanical	Compression	Cube	100 × 100 × 100	7, 14, 28, 56, 180	BS 12390-3^6^
	Splitting tensile	Cylinder	⌀100 × 200	7, 14, 28, 56, 180	BS 12390-6^61^
	Flexural	Prism	100 × 100 × 500	7, 14, 28, 56, 180	BS 12390-5^62,62^
	Modulus of elasticity	Cylinder	⌀100 × 200	28	ASTM C469 ^63^
	Ultrasonic pulse velocity	Prism	100 × 100 × 500	7, 14, 28, 56, 180	BS 12504-4^64,64^
	Drying shrinkage	Prism	75 × 75 × 290	1, 3, 7, 14, 21, 28, 56	ASTM C878/C878M-22^65^
Transport	Water absorption	Cylinder	⌀50 × 100	28	BS 1881-122 ^66^
	Sorptivity	Cube	⌀50 × 100	7, 28	ASTM C1403-15^67^
	Permeable porosity	Cylinder	⌀50 × 50	28	RILEM TC-14 CPC11^68^
Thermal	Conductivity	Rectangle	20 × 20 × 12	28	ASTM C177-19^69^
	Diffusivity	Rectangle	20 × 20 × 12	28	ASTM C177-19^69^
	Specific heat	Rectangle	20 × 20 × 12	28	ASTM C177-19^69^
Microstructural	SEM	Cube	15 × 15 × 15	28	ISO 16700^70^
	Mercury Intrusion Porosimetry (MIP)	Cube	15 × 15 × 15	28	ASTM D4404-18^71^

## Results and discussion

### Fresh concrete properties

#### Slump flow

**Figure 5:**
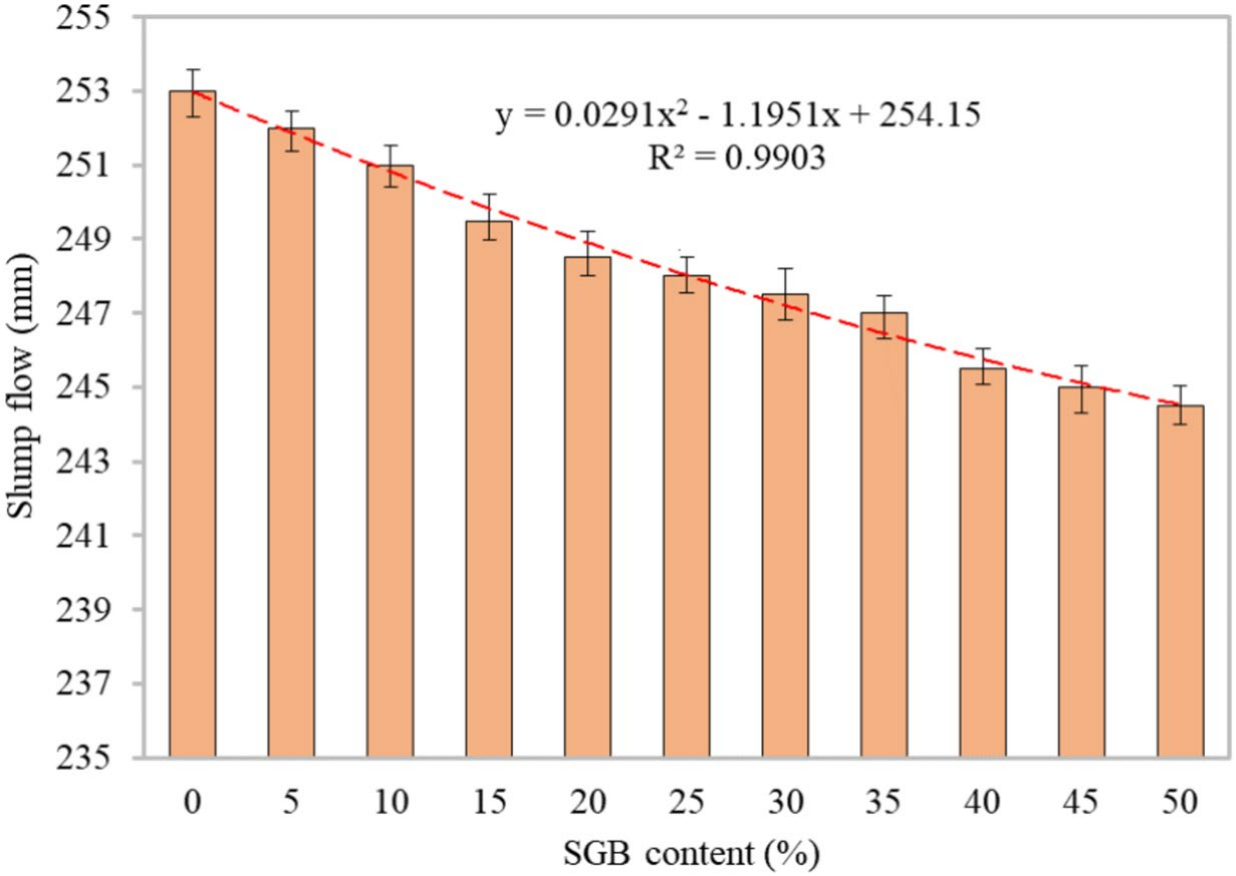
Slump flow of FC containing different percentages of SGB as sand replacement.

A slump test was conducted to analyse the FC’s flowability and fluidity^72^. The slump diameter in Fig. 5 determines the FC’s flowability. As the SGB’s weight fraction increased, the flow diameter decreased by 10 mm, 20 mm, 35 mm, 45 mm, 50 mm, 55 mm, 60 mm, 75 mm, 80 mm, and 85 mm compared to the control specimen, which recorded a flow diameter of 253 mm. The reduction in the slump flow was due to the irregular forms and sharper edges of the SGB particles compared to the sand particles, which led to a rise in friction and a reduction in the fluidity of concrete itself. Ahmad et al.^73^ mentioned a similar decrease in the workability. Substituting NFA with any waste material reduces the flow table’s spread diameter. A rise in water demands can account for a reduction in the fluidity, as the presence of the small particles of NFA or other waste substances necessitates a greater amount of water. According to Lim et al.^26^, the decrease in the slump flow may have a considerable impact on the mix, as determined by the surface characteristics of the waste glass grains. Additionally, an increase in the amount of waste glass in concrete results in a decrease in its compaction factor^40^.

#### Setting time

**Figure 6:**
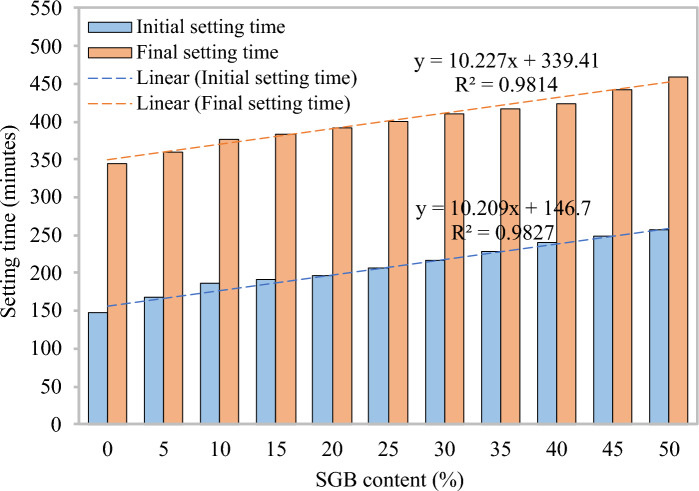
Initial and final setting times of FC containing different percentages of SGB as sand replacement.

Based on the results of three tests, Table 5 presents the mean, standard deviation, and coefficient of variation for the initial and final setting times. Figure 6 indicates that all the FC mixes had a significant increase in both the initial and final setting times. The control mix (S0) had an initial setting time of 148 min, while the S50 FC mixes had an initial setting time of 257 min. Due to the low water absorption of the SGB particles in the FC cementitious matrix, an increase in the SGB content prolonged the setting time^74^. Lu et al.^53^ reported a similar trend of delayed setting times with incremental glass powder use. The control mix (S0) had a final setting time of 344 min, whereas the S50 FC mixes experienced an increase to 459 min. The final setting time was increased by approximately 5, 10, 12, 14, 16, 19, 21, 23, 28, and 33%. The control mixes included 5%, 10%, 15%, 20%, 25%, 30%, 35%, 40%, 45%, and 50% of SGB as partial replacement in FC. The initial setting time ranged from 13 to 74%, while the final setting time ranged from 5 to 33%, with the replacement ratio of NFA by SGB increasing. Alyami et al.^75^ noted that the introduction of wastepaper sludge reduced the setting time. This was because the amorphous silica content delayed the hydration reaction, which in turn reduced the setting time and increased the replacement level. Additionally, the slow bonding of the SGB particles with the binder material resulted in a longer final setting time^76^.

**Table 5:** Values of mean, standard deviation, and coefficient of variation for initial and final setting times.

FC mix	Initial setting time			Final setting time		
	Mean	Standard deviation	Coefficient of variation (%)	Mean	Standard deviation	Coefficient of variation (%)
S0	148	3.61	2.44	344	2.65	0.77
S5	167	2.65	1.58	361	3.51	0.97
S10	187	2.65	1.41	377	2.00	0.53
S15	192	4.36	2.27	384	4.51	1.18
S20	196	3.21	1.64	391	5.00	1.28
S25	206	2.00	0.97	401	5.29	1.32
S30	217	2.65	1.22	410	2.00	0.49
S35	228	3.00	1.32	417	4.73	1.13
S40	240	3.61	1.50	424	3.79	0.89
S45	248	2.00	0.81	442	3.46	0.78
S50	257	4.51	1.76	459	2.00	0.44

#### Density

**Figure 7:**
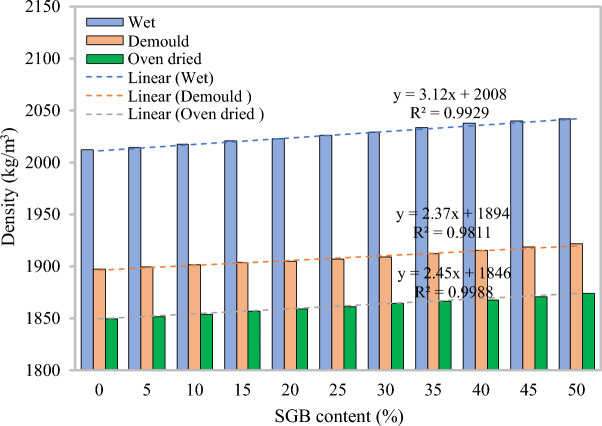
Density of FC containing different percentages of SGB as sand replacement.

Figure 7 displays the results for the wet and oven-dried SGB-blend FC mixes. The density of the oven-dried FC mixture increased from 1849 kg/m^3^ to 1874 kg/m^3^ as the SGB concentration increased from 0 to 50%. The FC’s fresh densities ranged from 2012 kg/m^3^to 2042 kg/m^3^, while the partial replacement of NFA with SGB ranged from 0 to 50%. Incorporating SGB resulted in a higher density than FC with NFA. SGB has a higher specific gravity than fine aggregate, which contributed to the increased density of FC mixes blended with SGB^37^. Hameed and Hamada^77^ similarly stated a rise in the FC’s density when the glass powder concentration increased. FC with the partial replacement of NFA with polyvinyl wastes had a density range of 1450 kg/m^3^ to 2000 kg/m^3^^33^. The density exhibited a positive correlation with the proportion of polyvinyl waste in the base mixture.

### Mechanical properties

#### Compressive strength

**Figure 8:**
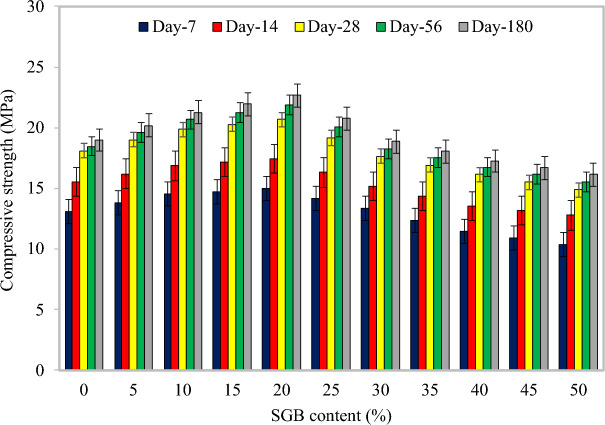
Compressive strength of FC incorporating different percentages of SGB as NFA replacement.

Figure 8 depicts the compressive strength results of FC with varying percentages of SGB as the NFA replacement at 7, 14, 28, 56, and 180 days. In comparison to the control FC, the FC’s compressive strength increased from 5.34% to 14.35%, 4.25% to 12.36%, 5.09% to 14.27%, 6.12% to 18.35%, and 6.43% to 19.39% after adding 5% to 20% SGB for 7, 14, 28, 56, and 180 days. A decrease in the compressive strength was then observed. The results indicated that replacing 25% of NFA with SGB resulted in higher compressive strength compared to the control FC after 7, 14, 28, 56, and 180 days. The increase in the compressive strength was attributed to two factors. Initially, CSH, calcium aluminium silicate hydrate, and additional hydration products were generated during the pozzolanic reaction of SGB^78^. Furthermore, the inclusion of SGB in concrete increased the density due to the infill effect, thereby influencing its strength parameter^79^. The compressive strength diminished when higher concentrations of SGB were present due to the adverse impacts of the contamination and workability^80^. The incorporation of polyvinyl wastes into FC resulted in the formation of the CSH gel, which improved the material’s compressive strength^34^. Sharipudin and Ridzuan^34^ stated that the FC’s compressive strength enhanced when RFA was partially substituted for NFA. Due to the pozzolanic properties of polyvinyl wastes, the formation of hydration products that facilitate hydration contributed to the density and mechanical properties of FC. Significant amounts of calcium oxide (CaO) were detected in polyvinyl waste, demonstrating that the highest level of reactivity was needed to generate CSH. By means of the pozzolanic reaction between SiO_2_ and CaOH, the replacement of RHA with NFA generated subordinate CSH, which contributed to the denser microstructure of FC^35^. Ramamurthy et al.^78^ provided evidence that the compressive strength of FC decreased as its density reduced, signifying that the dry density was a substantial factor in determining the FC’s compressive strength^81^.

#### Flexural strength

**Figure 9:**
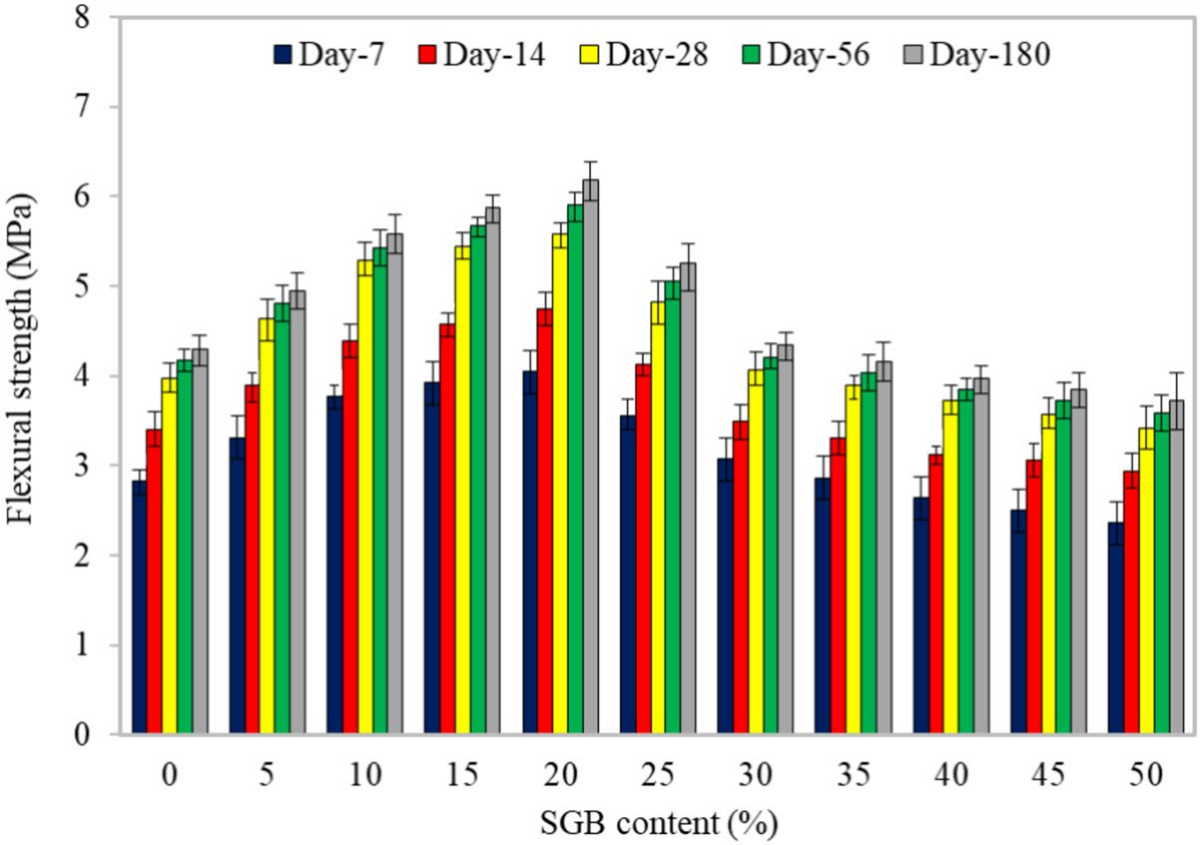
Flexural strength of FC incorporating different percentages of SGB as NFA replacement.

As shown in Fig. 9, FC was tested for the flexural strength at 7, 14, 28, 56, and 180 days following replacing NFA with SGB. The flexural strength increased from 16.8% to 42.84%, 14.47% to 39.36%, 16.77% to 40.83%, 15.07% to 41.44%, and 15.15% to 43.96% when compared to the control mix of FC with a dose of SGB ranging from 5 to 20%. As the number of days for curing increased, the FC’s flexural strength improved. The addition of SGB to FC enhanced the flexural strength by increasing the resistance between the constituents while establishing a stronger bond between them^82^. The hydration process in cement and SGB mixes resulted in an increased production of products derived from hydration. However, the dispersion of the SGB particles caused hydration products to harden, enhancing the mix’s flexural strength^83^. Mineral additives, when substituted for cement, enhanced the pozzolanic reactions and filling behaviours, leading to a long-term improvement in the strength^81^. The size of SGB was responsible for the increase in the flexural strength. For FC, the ratio of the flexural strength to the compressive strength was between 0.25 and 0.35. Glass with the particle sizes smaller than 75 mm exhibited the pozzolanic behaviour. A reduction in the particle size increased the glass’s pozzolanic behaviour^84^ due to the SiO_2_ interaction with calcium hydroxide (CH). Moreover, Hameed and Hamada^77^ reported that FC blended with glass powder had better flexural strength compared to FC blended with other forms of glass powder. Studies have also displayed the enhancement of the FC’s flexural strength through curing methods.

#### Splitting tensile strength

**Figure 10:**
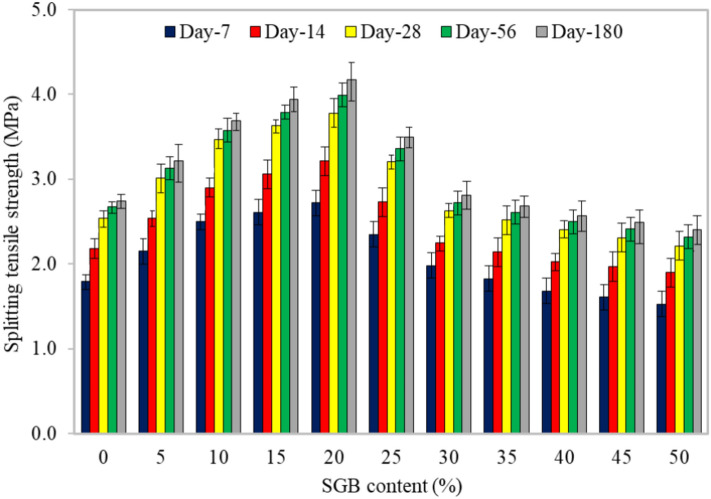
Splitting tensile strength of FC incorporating different percentages of SGB as NFA replacement.

There is a similar trend between the splitting tensile strength and flexural strength in FC mixes. Figure 10 illustrates the splitting tensile strength of 11 mixes cured for 7, 14, 28, 56, and 180 days. Equated to the reference mix, the splitting tensile strength development for the S5 mix decreased from 119.84% to 117.28% with an increase in curing time from 7 to 180 days. The splitting tensile strength development of the S5 mix declined from 119.84% to 117.28% when the curing age increased from 7 to 180 days, compared to the reference mix. The splitting tensile strength of the S10, S15, S20, S25, S30, S35, S40, S45, and S50 mixes rose from 139.24% to 134.43% compared to the reference mix. Similarly, for the same mixes, the splitting tensile strength increased from 145.48% to 143.5%. We observed the ranges of 130.99% to 127.48%, 110.59% to 103.38%, 93.87% to 93.69%, 89.74% to 90.69%, and 85.1% to 87.72% compared to the control mix. Increasing the amount of SGB resulted in a corresponding enhancement in the splitting tensile strength, with a potential rise of up to 30%. This was due to the increased shear capacity of the sand particles and binder paste^85^. Ahmad et al.^86^ reported that incorporating waste glass reduced the splitting tensile strength of concrete by 20%. Hameed and Hamada^77^ stated that the FC’s splitting tensile strength increased as the amount of glass powder increased, regardless of the curing processes used^77^. After 7 days, the splitting tensile strength ranged from 13.7% to 18.2% of the compressive strength. For the testing periods of 14, 28, 56, and 180 days, the splitting tensile strength ranged from 14.0% to 18.4%, 14.0% to 18.3%, 14.5% to 18.3%, and 14.5% to 18.4%, respectively. The SGB particles mixed with the cement-like matrix of FC during the hydration process, making CSH and calcium aluminate hydrate (CAH). The reactions mentioned above were critical for developing the mechanical properties and durability of FC. The CSH gel was responsible for providing the strength and rigidity, whereas the CAH gel improved the material’s resistance to chemicals and its durability performance^83^. In addition, the CSH gel occupied the empty spaces between the SGB particles, enhancing the overall strength and decreasing permeability, leading to a more resilient and enduring substance. The inadequate bonding between the SGB particles and hydrated cement paste was responsible for the observed strength decline when substituting 25–50% of sand with SGB^86^. When SGB replaced a higher percentage of sand, this bonding played a crucial role in determining the compressive failure. Furthermore, fine sand had superior fracture toughness when compared to SGB. These findings validated the results documented by Ramamurthy et al.^78^. According to Ibrahim et al.^87^ and Abdullah et al.^88^, the tensile and flexural strengths of FC should be between 0.35 and 0.15 times its strength under compression.

#### Modulus of elasticity

**Figure 11:**
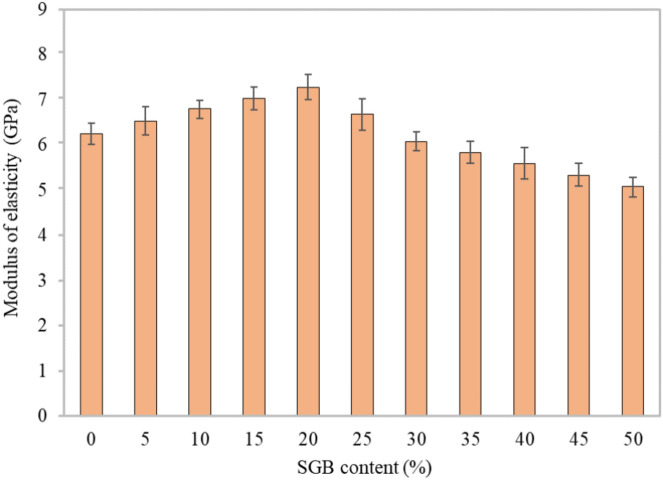
MOE of FC incorporating different percentages of SGB as NFA replacement.

Figure 11 demonstrates that after a 28-day curing period, SGB significantly affected the modulus of elasticity (MOE) of the FC mixes. MOE of the FC mixes depicted an upward trend as the proportion of SGB increased from 5 to 20%. It was indicated that MOE followed the same trend as the compressive strength. The inclusion of 20% aggregate replacement with SGB in FC resulted in an optimal MOE value of 7.22 GPa, which was approximately 16.43% higher than the control mix. As the density increased, both MOE and volume of the hydration products increased. The absence of coarse aggregate in the FC mixtures reduced MOE, a result of the enhanced consistency and evenness of the cement paste and mortar^7^. When NFA was filled, the air bubbles were coarse aggregates with varying strengths and MOE^89^. Compared to normal-weight concrete, FC’s MOE was noticeably lower. MOE can vary between 1000 and 8000 MPa, depending on the density at the dry stage, which ranged from 500 kg/m^3^ to 1500 kg/m^3^. Ramamurthy et al.^78^ revealed that conventional concrete had an MOE that was four times greater than FC. FC using FA exhibited a decreased MOE compared to FC containing NFA. This was due to a reduction in the NFA content and an increase in the binder paste content^90^. MOE of FC ranged from 1 kN/m^2^ to 12 kN/m^2^, depending on its dry density. The value was roughly 20% to 30% lower than MOE of conventional concrete^91^. Limbachiya et al.^44^ found that the inclusion of coarse waste glass decreased MOE.

#### Ultrasonic pulse velocity

**Figure 12:**
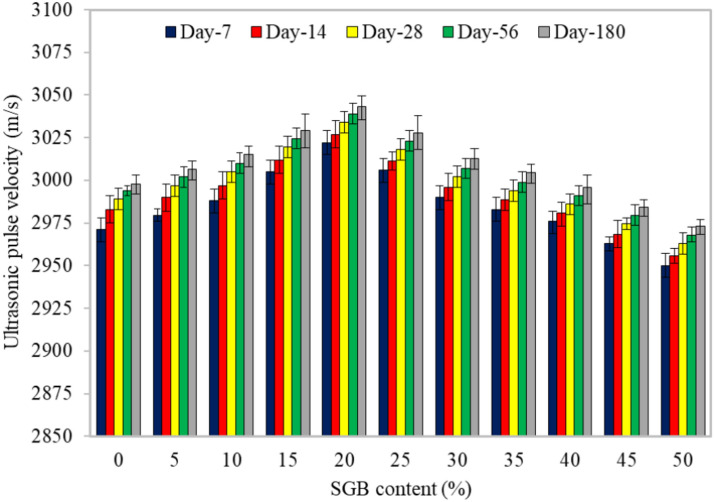
UPV of FC incorporating different percentages of SGB as NFA replacement.

Figure 12 shows the results of the ultrasonic pulse velocity (UPV) of FC incorporating different percentages of SGB as the NFA replacement. During the 7-day test, increasing the SGB content from 0 to 20% increased UPV from 2971 m/s to 3022 m/s. However, UPV then declined to 2950 m/s and continued to decrease by up to 50%. For 14, 28, 56, and 180 days, higher UPVs were observed as 3027 m/s, 3034 m/s, 3039 m/s, and 3043 m/s. All the FC mixes, across all the curing periods and SGB replacements, exhibited a modest increase. It was discovered that as the curing period increased and the SGB content increased up to 20%, the density and compressive strength rose while UPV dropped. All the FC mixes displayed a slight increase during all the curing periods and with every SGB replacement. It has been noted that as the curing period and SGB content increased up to 20%, the density and compressive strength also increased, but UPV dropped subsequently. The relationship between UPV and density was significant; denser materials with fewer pores provided higher UPV owing to the ease with which UPV travelled through the solid particles of FC^92^. As the curing period increased, UPV waves appeared denser and stronger^93^. Based on this study, incorporating SGB at a 30% rate improved the FC mixes’ quality while having little effect on their bubbles or pores, resulting in continuous voids. At the early stage of hydration, the transmitting time of UPV decreased with age, while UPV increased with age^94^. The influence of pores and their characteristics were estimated quantitatively on UPV. The pore radius, pore distribution, and porosity noticeably influenced UPV^95,96^.

### Transport properties

#### Sorptivity

**Figure 13:**
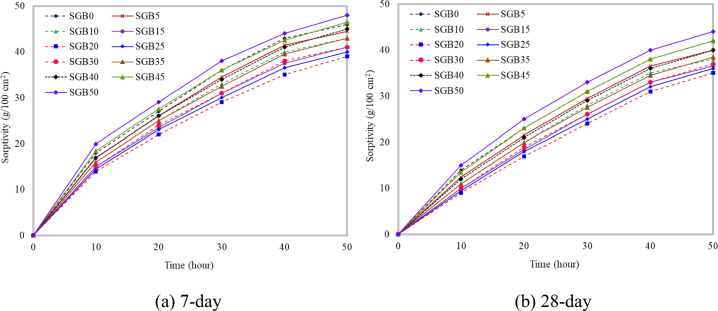
Sorptivity of FC incorporating different percentages of SGB as NFA replacement.

Figure 13a clearly illustrates that replacing some of NFA with SGB during the 7-day curing period led to a big drop in the sorptivity up to 20%. The increase in the SGB concentration resulted in the formation of hydration products, which in turn increased the FC’s density. Further increases in SGB decreased the sorptivity. However, the increase in the sorptivity was greater than that of the control FC. Entrained air spaces did not play a role in the transportation process; it mostly relied on the number of pores that contributed to capillary suction. The size of capillary pores decreased, resulting in a decrease in the sorptivity. The presence of numerous connected pores in FC enhanced its sorptivity characteristics. Figure 13b depicts that partial substitution of NFA with SGB over a 28-day curing period resulted in a maximum 20% decrease in the sorptivity. Nevertheless, the decrease in the sorptivity was more significant when compared to the 7-day period. The sorptivity rate increased as more hydration products were formed. However, the sorptivity demonstrated a larger increase compared to the control FC. An increase in the quantity of foam decreased the sorptivity^3^. The hygroscopic properties were assessed by measuring the sensitivity of FC. Ahamad and Chen^97^ reported that admixtures had an impact on the FC’s sorptivity outcomes. The sorptivity quantifies the water absorption in unidirectional FC^98^. The sorptivity features of the FC specimens indicated a remarkable increase during the early period, followed by a subsequent stabilization where they remained constant. The types of foaming agents, pore structures, mixture constituent features, and density all had a considerable impact on the sorptivity. A higher air content in FC increased the early sorptivity, especially when the air concentration exceeded 25%. Two clusters have been identified in FC, distinguished by their microstructure and components utilized^99^.

#### Water absorption

**Figure 14:**
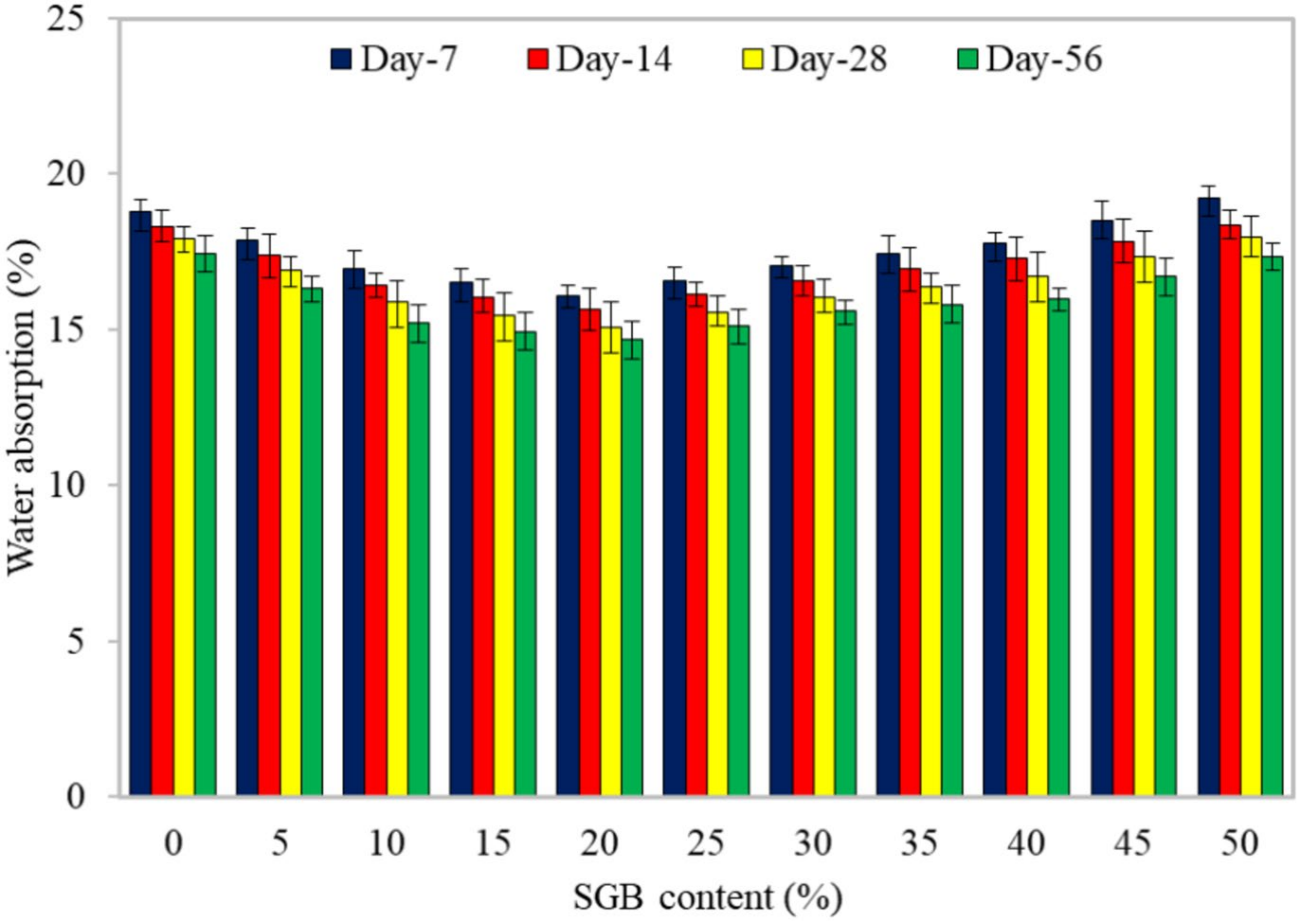
Water absorption of FC incorporating different percentages of SGB as NFA replacement.

According to Fig. 14, a longer curing period reduced the water absorption. An extended curing period produced extra hydration compounds, which expanded the density of FC and inhibited its water absorption. Furthermore, a corresponding rise in the density, reaching up to 20% of SGB when substituted for a portion of NFA in FC, resulted from the augmentation of the SGB content. The pozzolanic reaction of SGB, along with its filler effect on FC, contributed to the rise in the density. The S20 FC mix reduced the water absorption of about 14, 15, 16, and 15% throughout the 7-, 14-, 28-, and 90-day curing periods, respectively, as compared to the reference mix. The reference mix exhibited the minimum water absorption. However, replacing 50% of NFA with SGB resulted in higher water absorption compared to the control mix. The waste glass composition displayed a notable capacity for the water absorption^44^. The water absorption of FC showed an initial increase during the early immersion period, followed by a steady level^98^. A reduction in the volume of paste decreased the volume of capillary pores, subsequently reduced the density. The primary cause of this decline was a reduction in the FC’s water absorption. The paste phase, which discontinued all the artificial holes involved in the water absorption, primarily affected the water absorption of FC^81^. The number of pores and voids in FC directly correlated with the rise in the water absorption rates. The presence of connected pores in FC enhanced the water absorption. The quantity of the mixture paste also affected the water absorption. Essentially, a paste with a greater concentration absorbed a larger amount of water^100^.

#### Permeable porosity

**Figure 15:**
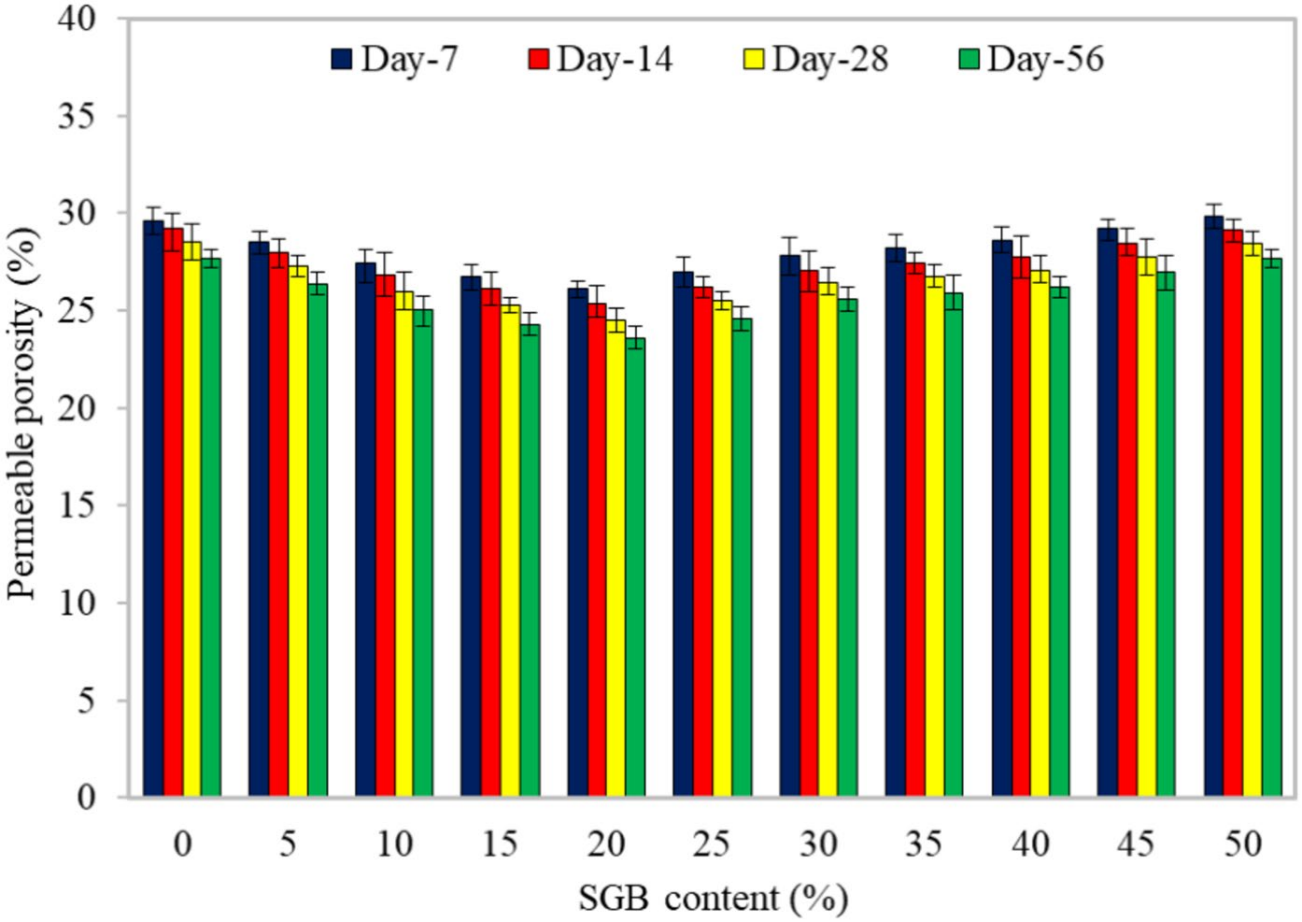
Permeable porosity of FC incorporating different percentages of SGB as NFA replacement.

Figure 15 shows an inverse relationship between the curing period and porosity, illustrating that longer curing times resulted in reduced porosity. As previously stated, prolonging the curing period led to a higher density of FC because pores reduced. Similar to the water absorption, substituting up to 20% of NFA in FC with SGB reduced the porosity. The porosity of the S20 mix decreased by approximately 12%, 13%, 14%, and 15% compared to the control mix during the 7, 14, 28, and 90-day curing periods, respectively. The porosity of FC is defined as the total volume of voids plus entrained air voids inside the paste^20^. Kearsley and Wainwright^20^ found a strong correlation between the porosity and dry density of FC. The FC’s porosity and pore structure primarily determined moisture or water transfer. The size of the pores and their degree of connection determined the permeability of FC^101^. As the density of FC increased, its permeability coefficient dropped, and its pores increased in the inverse proportion^102^. Kearsley and Wainwright^14^ evaluated the FC’s permeability and porosity using its density. FC had twice the water absorption of the control specimen, and its permeability was lower than that of the mix containing pulverized ash^103^. The porosity of FC did not solely determine its permeability^104^. The water-to-cement ratio and the hydration process caused the porosity in the mixture^105^.

#### Drying shrinkage

**Figure 16:**
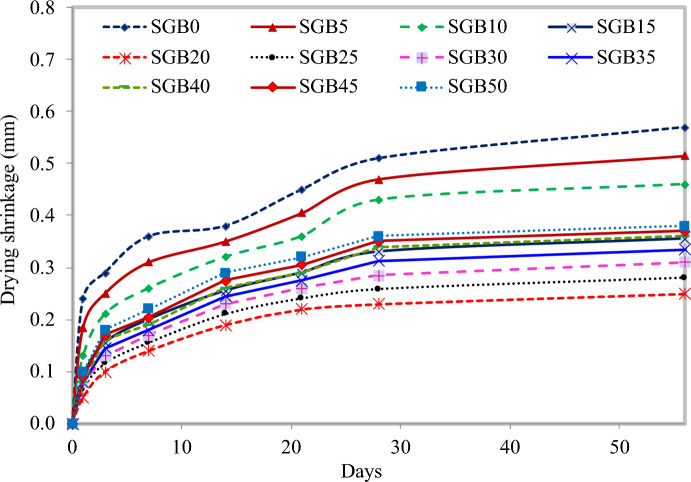
Drying shrinkage of FC incorporating different percentages of SGB as NFA replacement.

Figure 16 depicts that increasing the SGB percentage to 20% resulted in the most favourable drying shrinkage value. However, compared to the control FC and S10 mix, the drying shrinkage increase was somewhat less significant. Employing SGB as a partial replacement for NFA decreased the size of pores due to higher density. Drying shrinkage refers to the reduction in size of specimens, measured in one dimension, caused by the loss of moisture. In accordance with Wan et al.^106^, when the moisture content is low, the drying shrinkage of FC increases. Sun et al.^107^ discovered that the pore structure of FC could potentially explain the variations in the drying shrinkage of FC when using different foaming agents. By increasing the amount of FA used as a binder, drying shrinkage can be reduced when there is insufficient connection between pores^108^. The main cause of the drying shrinkage in FC is stress from the shrinkage owing to water loss from capillary pores. As the curing period progressed, there was an upward trend in the drying shrinkage from day 1, and the drying shrinkage subsequently remained stable after 28 days^109,110^.

### Thermal properties

#### Thermal conductivity

**Figure 17:**
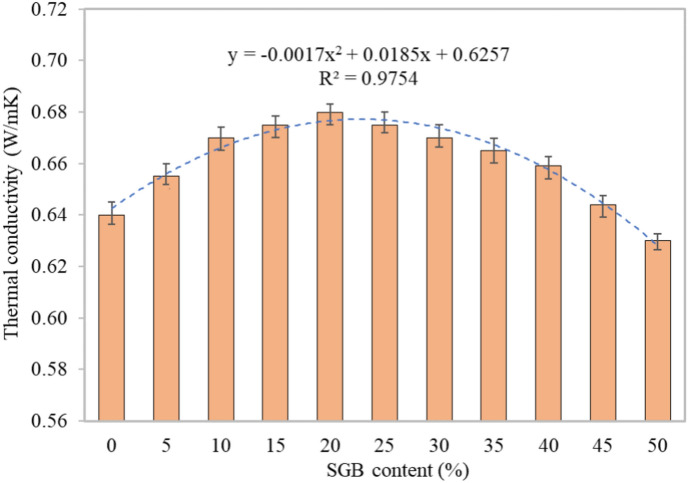
Thermal conductivity of FC incorporating different percentages of SGB as sand replacement.

Figure 17 demonstrates that the thermal conductivity of the control FC was 0.640 W/mK. However, when FC was substituted with 5%, 10%, 15%, and 20% SGB as a substitute for NFA, the thermal conductivity increased by 2.34%, 2.29%, 0.75%, and 0.74%, respectively, in comparison to the control specimen. The thermal conductivity peaked at a 20% replacement rate. The observed rise in the thermal conductivity with increasing the SGB replacement, up to a 20% level, can be attributed to the progressive formation of a denser solid structure with reduced empty spaces, hence enhancing the heat transfer. The formation of extra empty spaces inside the FC matrix appeared to be the cause of the observed decrease in the thermal conductivity, surpassing 20% of the SGB mixture. This trend aligned with the previously determined strength characteristics. Hameed and Hamada^77^ showed that increasing the amount of glass powder in FC mixes increased the thermal conductivity. Researchers found that the thermal conductivity of medium density (1000 kg/m^3^) was one-sixth of that of distinct cement mortars. The thermal conductivity of concrete exhibited an inverse relationship with its density^78^. When some of the binder was replaced with FA or a similar mineral admixture, the temperature and density went down because the hydration process did not make as much heat^85^. The use of low-density lightweight aggregates, along with intentionally including air voids in the mixture, increased the heat conductivity^111^. The increase in the thermal conductivity was owing to the FC’s reduced porosity^2^. The position and orientation of the pores influenced the thermal conductivity of FC. Factors such as aggregate types, density, pore size, mineral admixture, and foaming agent also had an impact^2^. When FA was used as a binding agent and subjected to elevated temperatures, it decreased the thermal conductivity^112^.

#### Thermal diffusivity

**Figure 18:**
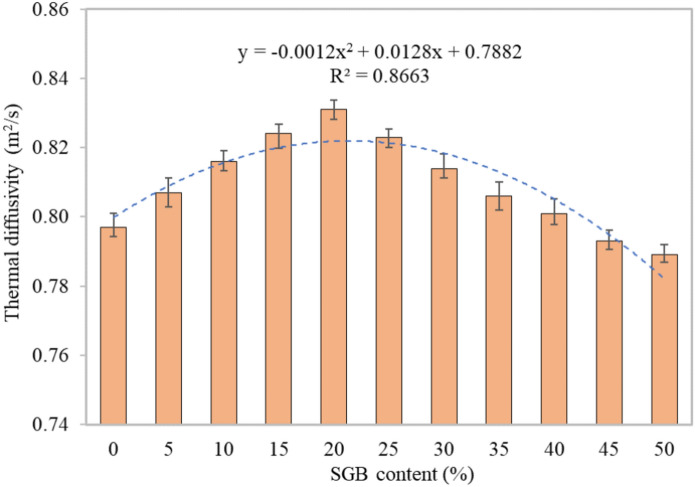
Thermal diffusivity of FC incorporating different percentages of SGB as NFA replacement.

Thermal diffusivity is the measure of the rate at which heat is transported from one substance to another due to a difference in temperature. The thermal diffusivity determines the relationship between the temperature inside the specimen and the temperature encountered on its surface during the procedure. Figure 18 displays that the S0 mix had a thermal diffusivity of 0.797 m^2^/s, the S20 mix had the highest at 0.831 m^2^/s, and the S50 mix had the lowest at 0.789 m^2^/s. The thermal diffusivity values for S5, S10, S15, S20, S25, S30, S35, S40, S45, and S50 appeared in an increasing direction. The values were roughly 101.25%, 102.38%, 103.39%, 104.27%, 103.26%, 102.13%, 101.13%, 100%, 99.5%, and 99%, respectively. These values were related to the control concrete mix (S0). The increase in the thermal diffusivity seen in the FC specimens with a 20% SGB replacement can be attributed to the decrease in open voids in the microstructure. This reduction allowed for more efficient heat transmission because the microstructure was denser. The higher the impermeable porosity of the FC sample, the more efficient the heat transmission over the entire matrix. Bilski et al.^112^ reported the impact of the density on the calculation of the thermal diffusivity coefficient. The relationship between the bulk density and thermal diffusivity indicated the existence of a one-way heat transmission equation^112^. According to Mydin^113^, replacing NFA in FC with lightweight aggregate improved the diffusivity. An increase in the quantity of NFA in the FC decreased the diffusivity^114^.

#### Specific heat capacity

**Figure 19:**
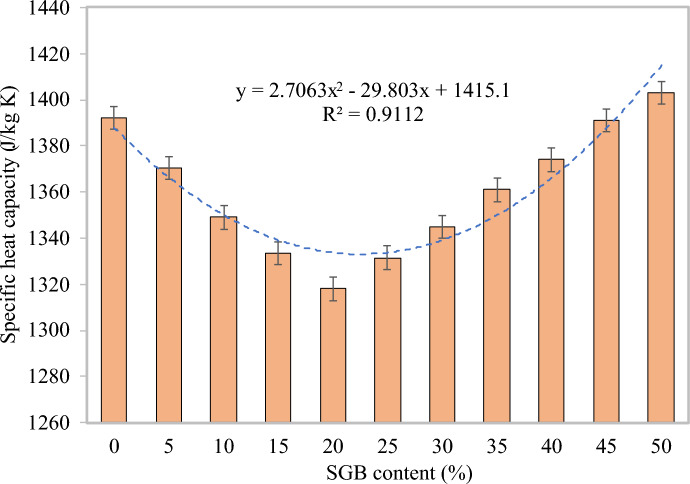
Specific heat capacity of FC incorporating different percentages of SGB as NFA replacement.

Thermal diffusivity and specific heat capacity establish the heat transfer in FC. The S0 mix had a specific heat capacity of 1392 J/kgK, the S20 mix had the lowest specific heat capacity at 1318 J/kgK, and the S50 mix had the highest specific heat capacity at 1403 J/kgK. The specific heat capacity for the S5, S10, S15, S20, S25, S30, S35, S40, S45, and S50 mixtures, as presented in Fig. 19, ranged from 98.46% to 100.79% of the control FC (S0). Based on Mydin^113^, increasing the amount of lightweight aggregate in NFA in FC beyond the optimum proportion increased the specific heat. The higher water absorption capability of the lightweight aggregate accounted for this. Fu and Chung^115^ noted that increasing the amount of latex in FC decreased the specific heat. An increase in the FC’s density decreased the specific heat^114^. Utilizing mortar with a higher compressive strength results in enhanced specific heat and conductivity. A decrease in the FC’s permeable porosity decreased the specific heat value^114^.

There is influence of the SGB-blended FC on the thermal diffusivity and specific heat capacity compared to traditional FC was reported in the thermal properties section. Application of these kinds of materials in energy-efficient buildings will reduce their thermal properties, which can be recommended for engineers and researchers.

### Microstructural assessment

#### SEM analysis

**Figure 20:**
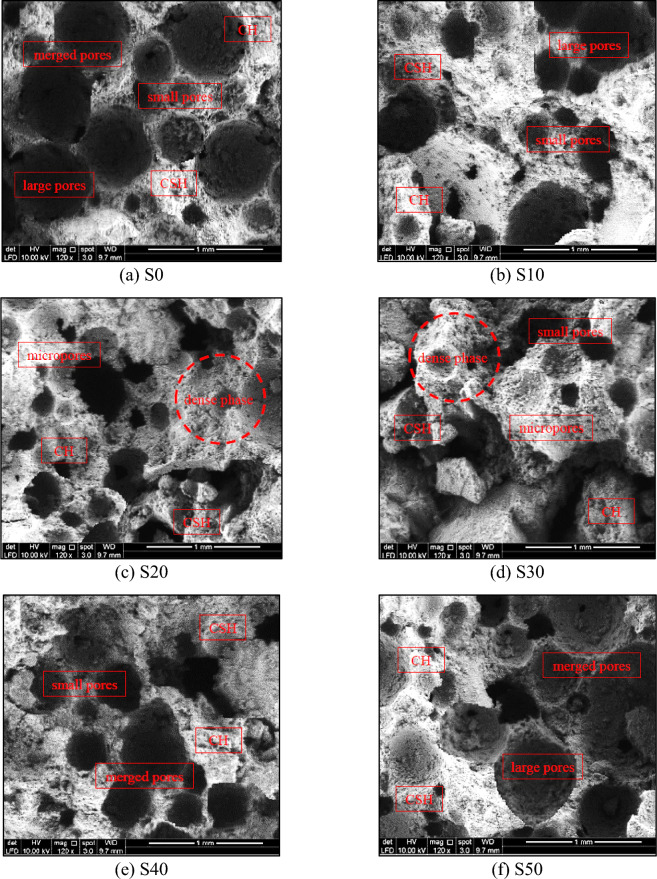
SEM analysis of FC incorporating different percentages of SGB as NFA replacement.

Figure 20 depicts the SEM analysis of FC using different amounts of SGB as a substitute for NFA. The results are illustrated for six different mixtures, specifically S0, S10, S20, S30, S40, and S50, for the purpose of comparison. The S20 mix (Fig. 20c) exhibits pores with a diameter less than 200 µm, resulting in the increased density and enhanced FC’s characteristics. Conversely, the control FC mix (S0) (Fig. 20a) had the largest pore size. The darker regions in the SEM images indicate the presence of capillary holes, while the formation of white crystals confirms the presence of hydration products. The control mix comprised fused pores, leading to a matrix with the reduced density. The S10 mix (Fig. 20b) had a moderate pore size, which accounted for its superior FC’s properties in comparison to the reference mix. The decrease in the pore size was a result of the synthesis of substances during the hydration and filling effect of SGB in FC. Consequently, the production of CH decreased. When comparing the S20 mix to the S30 mix, the pore size of the S30 mix showed a greater range, as seen by the SEM image (Fig. 20d).

When SGB was present in the FC cementitious matrix, it caused a breakdown of parts of the CH crystals that had high levels of calcium throughout their interactions with SGB. Additionally, the large specific surface area of SGB acted as the basis for the creation of the CSH gel and promoted the growth of more CSH gel on its surface. As a consequence, this reduced both the thickness and porosity of the interfacial transition zones (ITZs). Due to the greater porousness of ITZ compared to the bulk paste, SGB acted as a more efficient form in these porous conditions, therefore, facilitating the creation of the CSH gel.

Figure 20e demonstrates that the S40 mix had an apparent increase in voids larger than 1000 µm. Furthermore, as shown in Fig. 20f, the S50 mixture increased the size of these larger pores. One visible effect of the expansion of the pores was a decrease in their mechanical properties. According to Hadipramana et al.^29^, the smaller non-pozzolanic particles and smaller pozzolanic particles in FC fill the pores, resulting in an increase in the density. Sun et al.^107^ verified the influence of the thickness of the walls between pores, size of the pores, and connectivity of the pores on both the mechanical and transport properties. They conducted this verification by analysing a SEM image. Using SEM, Amran and Fazadnia^7^ also observed numerous spherical pores of varying sizes in FC. Images obtained from SEM facilitate the observation of the formation of CSH, CH, and ettringite products that occur during the FC’s hydration. The average pore diameter, pore dispersion, and porosity had a greater impact on the FC’s characteristics^116^. An increase in the porosity due to a higher quantity of SGB, which transitions into a less dense state, will result in an escalation in the water absorption. Because of its compact and uniform nature, the dense microstructure of FC reduces the presence of voids^117^. The SEM microstructure analysis clarifies the influence of NS on its rheological, strength, and durability characteristics. NS not only fills the pores in FC but also stimulates the formation of CSH to fill the pores, resulting in a dense microstructure. The absence of NS during crystal formation results in the growth of larger crystals and creation of larger pores. However, incorporating NS leads to more densely packed crystals with smaller pores. These small crystals form connections with each other, resulting in the increased density and a more compact microstructure.

ITZ between the aggregates (fine sand and SGB) and cementitious matrix is often a vulnerable area in porous FC. ITZ plays a pivotal function in the microstructure of FC which affected its mechanical and durability properties. ITZ is located between aggregates and cementitious paste in FC. It experiences structural alterations as a result of the existence of aggregates, which might potentially weaken the FC’s strength and durability properties. The formation of ITZ is a consequence of the wall effect which arises from the disparity in size between the cement particles varying from less than 1 micron to 80 μm and the significantly larger aggregate particles. The variation in size causes an alteration in the structure of the cement particles, resulting in “wall effect”. The optimal characteristics for SGB aggregates in FC were the crystalline and rougher surface. SGB exhibits brittleness due to its amorphous structure, but when it undergoes a transformation to a more crystalline shape with an ordered arrangement, it becomes less brittle. The coarse texture of SGB aggregates facilitated enhanced adhesion with the cement paste thus plays an important role to enhance the strength properties of FC as well as its durability performances. Employing SGB as a partial substitute for NFA in FC resulted in a packed and homogeneous morphology that displayed excellent bonding with the surrounding paste.

Additionally, the micro-hardness of ITZ between the cement paste and SGB remained intact, resulting in the creation of smaller mesopores and macropores, as observed in the SEM analysis of the microstructure. In contrast, the sample made with NFA alone experienced dissociation. ITZ of SGB-modified FC is constricted, with a decreased number of pores, and does not contain a higher concentration of CH crystals either within or near ITZ. Utilizing SGB as a partial substitute for sand in FC led to a compact and consistent structure that exhibited strong cohesion with the enclosing paste. There were no discernible differences in the overall structure between the two components.

#### Pore distribution

**Figure 21:**
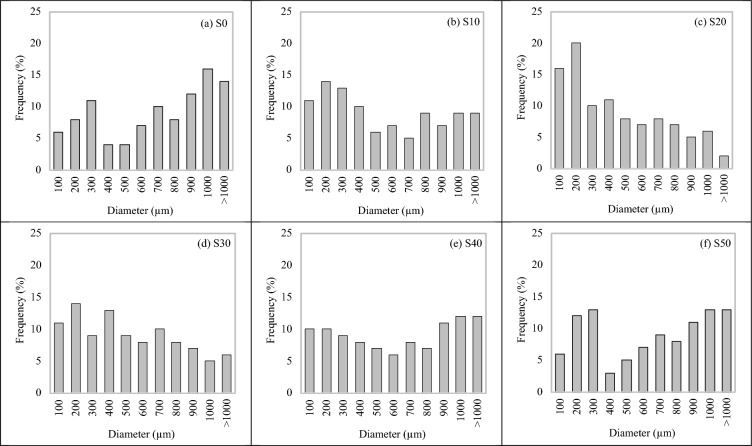
Pore distributions of FC incorporating different percentages of SGB as NFA replacement.

Figure 21 depicts the distribution of pores in FC with different proportions of SGB used as a replacement for NFA. For comparison, the studies only present the results for six different mixtures as S0, S10, S20, S30, S40, and S50. The frequency distribution of the pore diameters in the control specimens differed from that of the reference specimen, as illustrated in Fig. 21a. The count of pores with a size exceeding 1000 µm was recorded, indicating a significantly low density. On the other hand, Fig. 21b shows a decrease in frequency for the S10 mix. Based on Fig. 21c, it is evident that the quantity of pores with sizes smaller than 200 µm was higher, while the quantity of pores with sizes larger than 1000 µm was lower, in comparison to the other mixtures. This disparity contributed to the enhancement of the mechanical, transportation and thermal properties, as discussed in the earlier sections. However, it can be observed from Figs. 21d and e that there was a drop in the number of pores with a size smaller than 200 µm and an increase in the number of pores with a size larger than 1000 µm.

**Figure 22:**
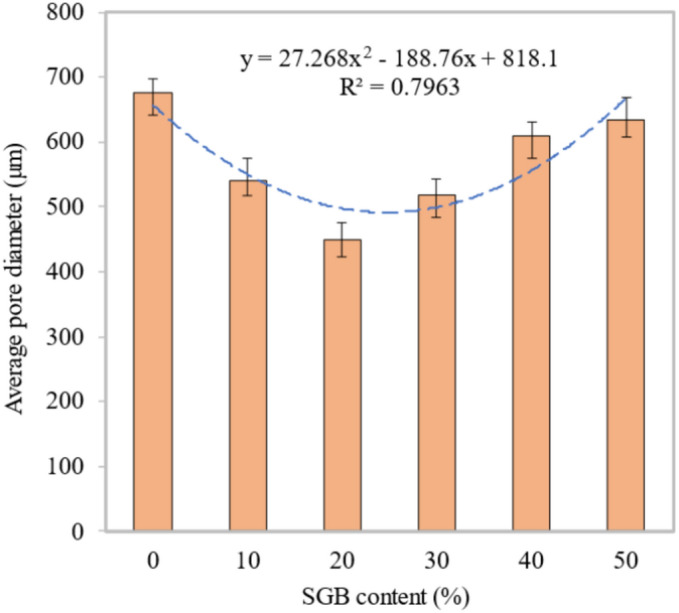
Average pore diameter of FC incorporating different percentages of SGB as NFA replacement.

**Figure 23:**
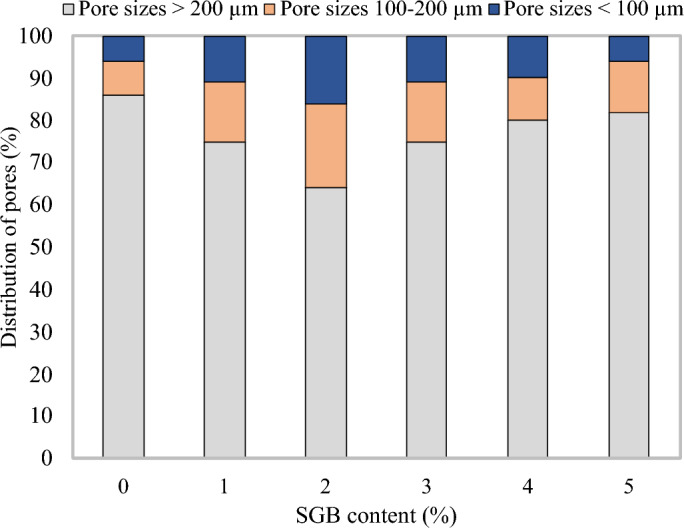
Percentage distribution of fine capillary, large capillary, and large pores of FC incorporating different percentages of SGB as NFA replacement.

This leads to unfavourable mechanical, transport, and thermal properties, as explained in the preceding sections. As can be seen from Fig. 22, the average pore diameters for the FC mixes with 10%, 20%, 30%, 40%, and 50% SGB as replacement for NFA were approximately 675 m, 540 m, 449 m, 518 m, 610 m, and 634 m. The S10, S20, S30, S40, and S50 FC mixes had pore size diameters of 80.00%, 66.52%, 76.74%, 90.37%, and 93.93% of the control mix, respectively. Figure 23 displays the distribution of pore sizes in FC blend with SGB. The S20 mix demonstrated a higher number of pores with sizes smaller than 100 µm and between 100 µm and 200 µm. These additional pores enhanced the FC’s fresh and hardened qualities. RHA in FC decreases the porosity and quantity of CaOH in ITZ^38^. RHA reduces the width of ITZ between NFA and cement paste^118^. The expansion of the pore diameter leads to a reduction in the strength properties of the lower density (less than 1000 kg/m^3^) of FC. However, the alignment of the binder paste governs the strength properties at higher densities (more than 1000 kg/m^3^)^119^. The regularity of the pore distribution in FC has an impact on its various characteristics. A tested sample with a high degree of pore distribution uniformity results in enhanced mechanical properties^107^. In FC, the thicker pore wall structure, smaller pores, and fewer connections between pores all work together to improve its mechanical properties and lower its water absorption^107^. Over half of the sizes within the air spaces are smaller than 200 µm. A SEM image intricately links the distribution of the FC’s pore size to the substitution of cement using pozzolanic materials^108^.

### Correlation between properties

Tam et al.^120^ mentioned that for FC, there was an upsurge in the strength parameter when the water-to-binder ratio was larger. This represents a deviation from the typical pattern found in traditional concrete. Most of the correlations found in the literature demonstrated the relationship between the compressive strength and several other mechanical parameters. To minimize expenses, save natural resources, and mitigate environmental damage associated with predicting material qualities, a majority of researchers^121–123^ have advocated for utilizing statistical and machine learning techniques to predict these properties.

#### Flexural–compressive strengths relationship

**Figure 24:**
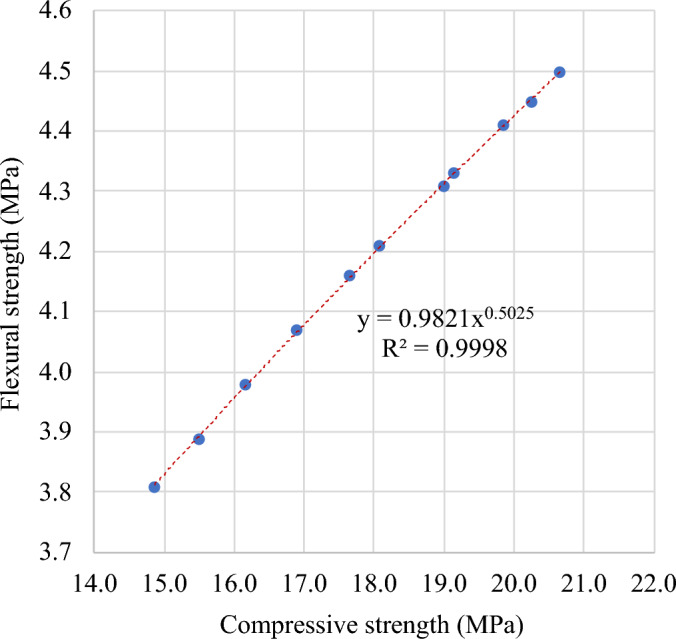
Correlation between flexural and compressive strengths of FC incorporating different percentages of SGB as NFA replacement at 28 days.

Figure 24 depicts how a power equation links the flexural strength and compressive strength of FC with SGB after 28 days of curing. Equation (1) illustrates the correlation between flexural strength and compressive strength, exhibiting a coefficient of determination (R^2^) of 0.99.

1 $$f_{f} = 0.0529 \sqrt {f_{c} }$$

Table 6 summarizes the correlation between the compressive strength and flexural strength, as represented by Eqs. (2) and (7). According to Yusuf et al.^124^, most of the relationships in the literature and international standards are 0.5 power relationships. From Fig. 24, it is noted that Eq. (1) displays a higher prediction or a prediction nearer to the actual experimental results. A lower prediction is observed for Eq. (6), which is nearer to Eq. (5).

**Table 6:** Correlation between compressive and flexural strengths.

Reference and Year	Relationship	Equation
Mydin et al. 2023^123^	$f_{f} = 0.2101 f_{c} + 0.0563$	(2)
Ahmed et al. 2008^125^	$f_{f} = 0.68 \sqrt {f_{c} }$	(3)
IS 456: 2000^126^	$f_{f} = 0.70 \sqrt {f_{c} }$	(4)
ACI 318: 2019^127^	$f_{f} = 0.62 \sqrt {f_{c} }$	(5)
NZS-3101: 1995^128^	$f_{f} = 0.60 \sqrt {f_{c} }$	(6)
EC-02: 2004^129^	$f_{f} = 0.201 f_{c}$	(7)

**Figure 25:**
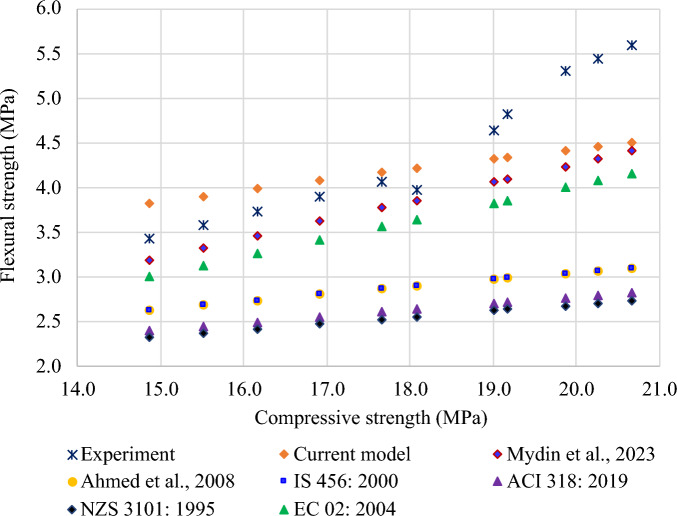
Validation of model with respect to previous models from literature.

Validation of the model is presented in Fig. 25 using equations from literature and international standards, with the help of the results from the experiments.

#### Splitting tensile–compressive strengths relationship

**Figure 26:**
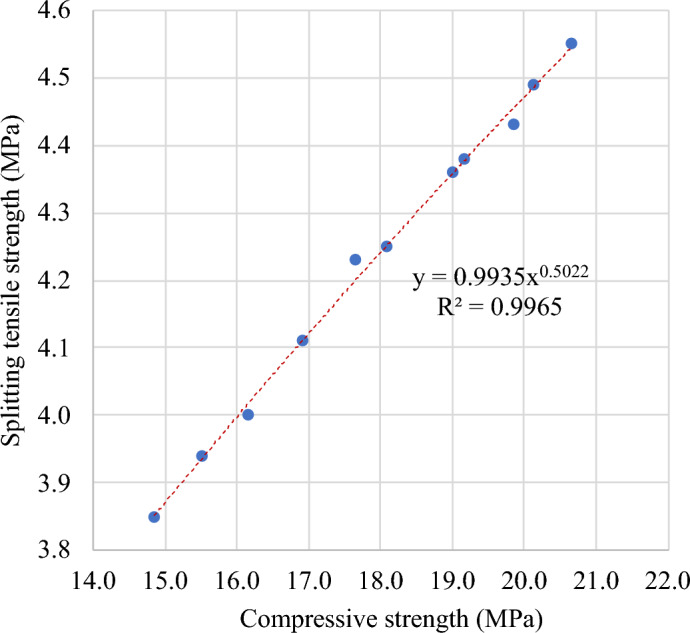
Correlation between splitting tensile and compressive strengths of FC incorporating different percentages of SGB as NFA replacement.

A strong correlation was witnessed between the splitting tensile strength and compressive strength of FC with SGB after 28 days, as shown in Fig. 26. Equation (8) indicates a high connection (R^2^ = 0.99) between the splitting tensile strength and compressive strength of FC containing SGB.

8 $${f}_{t}=0.9893 \sqrt{{f}_{c}}$$

**Figure 27:**
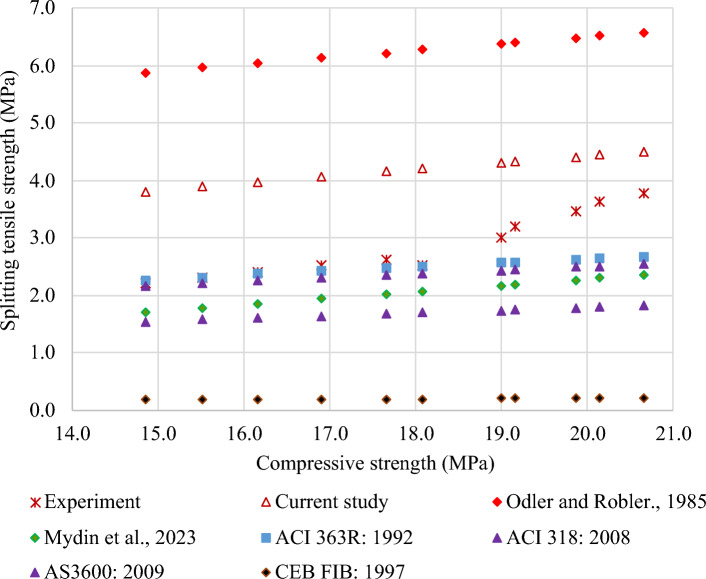
Validation of model with respect to previous models from literature.

The relationship between the splitting tensile strength and compressive strength of FC is demonstrated in Table 7, as reported in the literature and international standards. Table 7 comprises multiple models sourced from the literature, encompassing Eqs. (9) to (14). Most of the interactions discussed in the literature and standards revolve around a power dynamic. The proposed model was verified by comparing it with established models from standards and available literature, as illustrated in Fig. 27. The model proposed by Odler and Robler^130^ offers a more accurate estimation of the splitting tensile strength of FC. The CEB-FIB: 1997 model provides a cautious approximation of the splitting tensile strength of FC.

**Table 7:** Correlation between splitting tensile and compressive strengths of FC.

Reference and year	Relationship	Equation
Odler and Robler 1985^130^	$f_{t} = 0.12 f_{c} + 4.1$	(9)
Mydin et al., 2023^123^	$f_{t} = 0.1107 f_{c} + 0.0664$	(10)
ACI 363R: 1992^132^	$f_{f} = 0.59 \sqrt {f_{c} }$	(11)
ACI 318: 2019^127^	$f_{f} = 0.56 \sqrt {f_{c} }$	(12)
AS 3600: 2009^133^	$f_{f} = 0.40 \sqrt {f_{c} }$	(13)
CEB-FIB: 1997^131^	$f_{f} = 0.30 f_{c}^{0.66}$	(14)

#### Modulus of elasticity–compressive strength relationship

**Figure 28:**
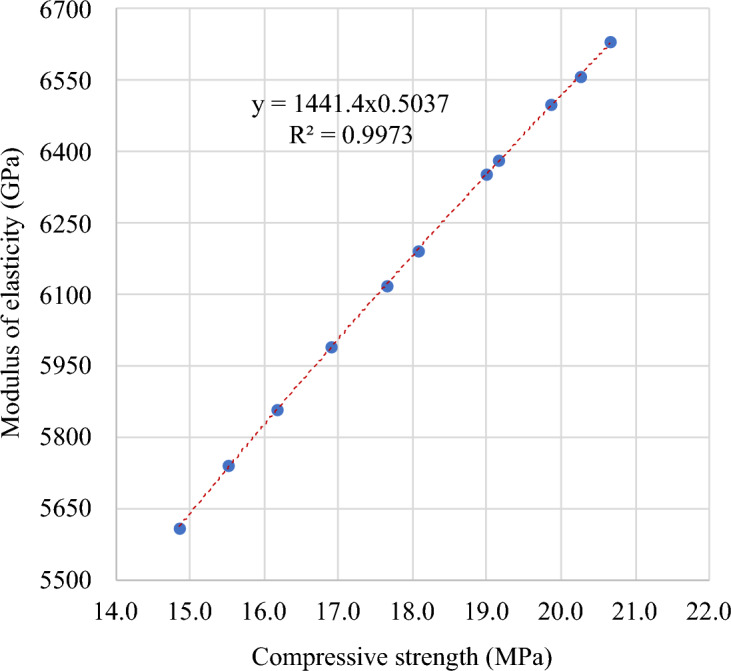
Correlation between MOE and compressive strength of FC incorporating different percentages of SGB as NFA replacement.

A power correlation was identified between MOE and compressive strength of FC with SGB at 28 days. This relationship is depicted in Fig. 28. Equation (15) indicates a strong correlation (R^2^ = 0.99) between MOE and compressive strength of FC with SGB:

15 $${f}_{t}=1441.4 \sqrt{{f}_{c}}$$

**Figure 29:**
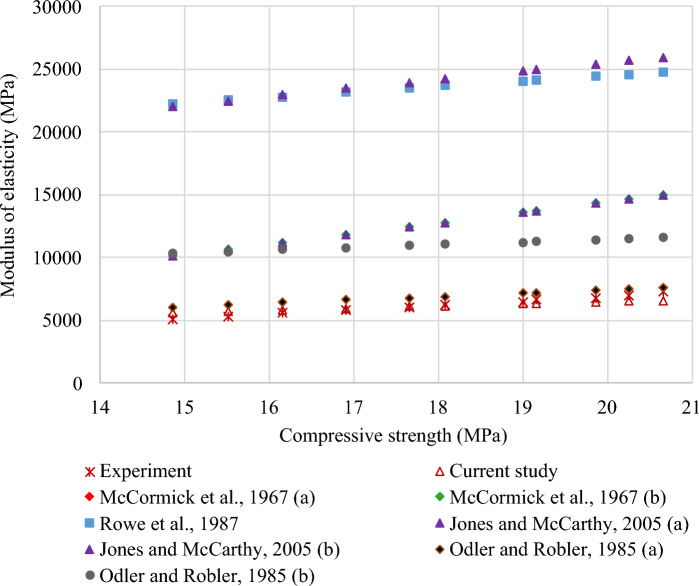
Validation of model with respect to previous models from literature.

The relationship between MOE and compressive strength of FC is presented in Table 8 based on the literature and international standards. Table 8 includes various models from the literature, including Eqs. (16) and (23). Most of the relationships discussed in the literature and standards were founded on the power relationship. The proposed model was validated by comparing it with the established models from standards and literature, as displayed in Fig. 29. The projected model has a higher degree of consistency with the model proposed by Older and Robler^130^. The predicted MOE, as established by Rowe et al.^136^ and Jones and McCarthy^85^, is larger than the actual experimental result.

**Table 8:** Correlation between MOE and compressive strength of FC.

Reference and year	Relationship	Equation
McCormick et al. 1967^134^	$E_{s} = 990 f_{c}^{0.67}$	(16)
McCormick et al. 1967^135^	$E_{s} = 420 f_{c}^{1.18}$	(17)
Rowe et al. 1987^136^	$E_{s} = 9100 f_{c}^{0.33}$	(18)
Kamara et al. 2008^137^	$E_{s} = 5700 \sqrt {f_{c} }$	(19)
Jones and McCarthy 2005^85^	$E_{s} = 420 f_{c}^{1.18}$	(20)
Jones and McCarthy 2006^90^	$E_{s} = 990 f_{c}^{0.67}$	(21)
Odler and Robler 1985^130^	$E_{s} = 1845 f_{c}^{0.59}$	(22)
Odler and Robler 1985^130^	$E_{s} = 217 f_{c} + 7090$	(23)

#### Compressive strength–UPV relationship

**Figure 30:**
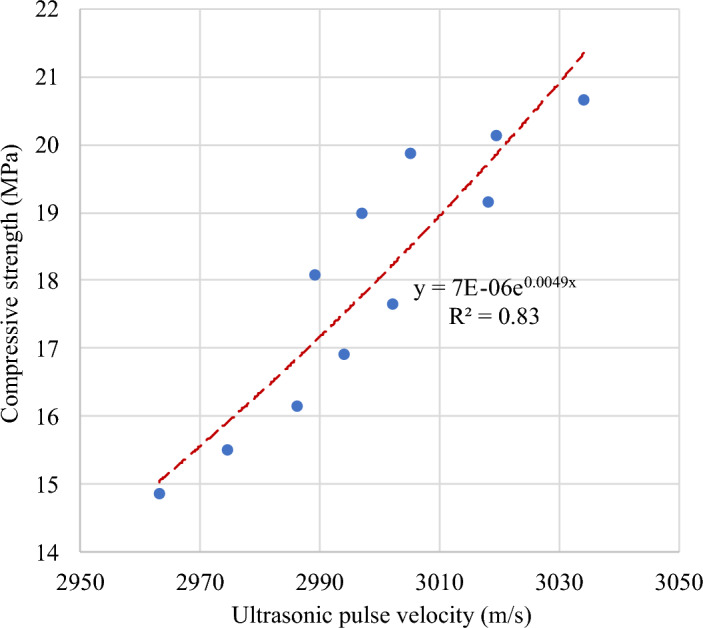
Correlation between UPV and compressive strength of FC incorporating different percentages of SGB as NFA replacement.

Figure 30 demonstrates a strong exponential correlation between the compressive strength and UPV of FC with SGB at 28 days. Equation (24) exhibits the correlation between the compressive strength and UPV of FC with SGB, having an R^2^ value of 0.83.

24 $${f}_{c}=0.000006 {e}^{0.005 V}$$

**Figure 31:**
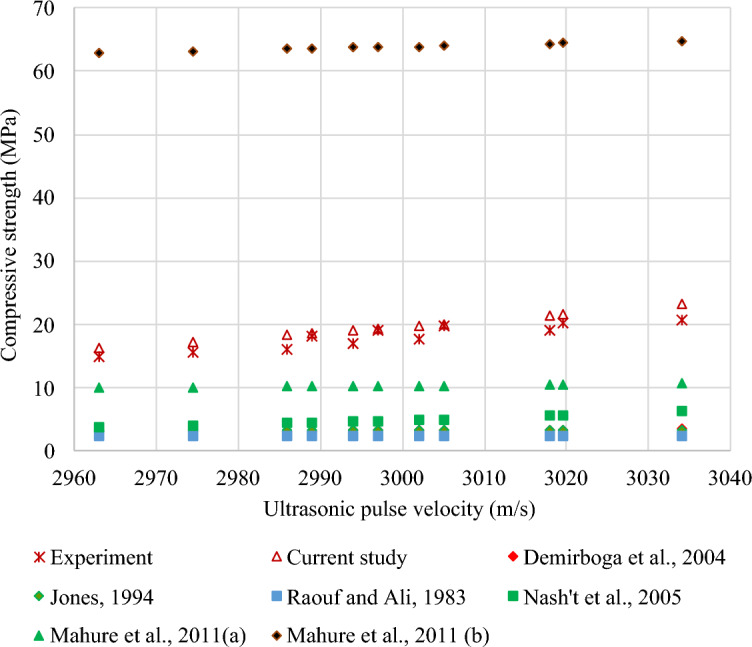
Validation of model with respect to previous models from literature.

The relationship between the compressive strength and UPV of FC is indicated in Table 9. Table 9 comprises a collection of various models referenced in the literature, namely Eqs. (25) to (29). Most relationships discussed in the literature illustrated an exponential trend. The expected compressive strength of FC, as stated by Nash’t et al.^141^, indicates a similar level of accuracy in both experimental and modern models. Mahure et al.^142^ reported a higher level of accuracy in predicting the compressive strength of FC compared to the model proposed by Raouf and Ali^140^, which produces lower estimates. The proposed model was validated by comparing it with the existing models from the literature, as depicted in Fig. 31.

**Table 9:** Correlation between compressive strength and UPV of FC.

Reference and year	Relationship	Equation
Demirboga et al. 2004^138^	$f_{c} = 0.008 e^{0.002 V}$	(25)
Jones 1962^139^	$f_{c} = 2.8 e^{0.000053 V}$	(26)
Raouf and Ali 1983^140^	$f_{c} = 2.016 e^{0.000061 V}$	(27)
Nash’t et al. 2005^141^	$f_{c} = 1.19 e^{0.0715 V}$	(28)
Mahure et al. 2011^142^	$f_{c} = 0.009502V - 18.19$	(29)

## Conclusions

Utilizing discarded SGBs as a replacement for NFA in manufacturing of FC has become a potential method to encourage sustainable construction practices. This technique offers a dual advantage by effectively channelling glass waste away from landfills and reducing the demand for natural sand resources. Incorporating pulverized and refined glass powder into the FC mixture preserves the structural and transportation characteristics while simultaneously promoting environmental sustainability. The conclusions drawn from this investigation are as follows:

- The inclusion of SGB as a partial substitute for NFA led to a prolongation of the setting times. The FC mixes containing 0% and 50% SGB had final setting times of 344 and 459 min, respectively. Replacing the NFA content with SGB reduced the workability of the FC mixes. This resulted in the slump flow diameters of 253 mm and 244 mm for the FC mixtures with 0% and 50% SGB, respectively.
- The inclusion of greater SGB content slightly increased the density of FC. The greater specific gravity of SGB contributed to the increase in the density of the FC mixes when combined with it. By using foam and SGB as substitutes for 0% (control) to 50% of sand, it was possible to create FC mixtures with a uniform density, maintaining a density within the range of 1850 ± 25 kg/m^3^.
- The use of SGB enhanced the FC’s strength properties. The FC mixture with 20% SGB exhibited the compressive, flexural, and splitting tensile strength values of 20.7 MPa, 5.58 MPa, and 3.78 MPa, respectively, after 28 days.
- The inclusion of SGB improved the FC’s transport capabilities. FC with a 20% SGB content demonstrated the lowest sorptivity. Specifically, after 50 h of testing, the sorptivity measured 39 g/100 cm^2^ at 7 days of age and 35 g/100 cm^2^ at 28 days of age.
- As displayed by SEM, the addition of 20% SGB as a replacement for sand reduced the number of voids larger than 600 μm. It also decreased the diameter of the voids and improved their homogeneity.
- Using the modelling techniques, FC-SGB results were compared to prior investigations in terms of the mechanical properties. In assessing the experimental data against the predictions of the mechanical characteristics using various models, the results indicated a strong correlation between the strength properties and predictions.

## Data Availability

The datasets used and/or analysed during the current study are made available from the corresponding authors on reasonable request.

## Abbreviations

FC: Foamed concrete
SGB: Soda-lime glass bottle
NFA: Natural fine aggregate
FA: Fly ash
POFA: Palm oil fuel ash
RHA: Rice husk ash
RFA: Recycled fine aggregate
CSH: Calcium silicate hydrate
*E*_s_: Static modulus of elasticity
*f*_*c*_: Compressive strength of concrete
*f*_*t*_: Splitting tensile strength of concrete
*t*: Age of specimen
*p*: Porosity
